# Supplementary figures and images for: Systematic characterization of plant-associated bacteria that can degrade indole-3-acetic acid
Source: PLoS Biol. 2024 Nov 26;22(11):e3002921. doi: 10.1371/journal.pbio.3002921 (PMC11630574; doi:10.1371/journal.pbio.3002921)

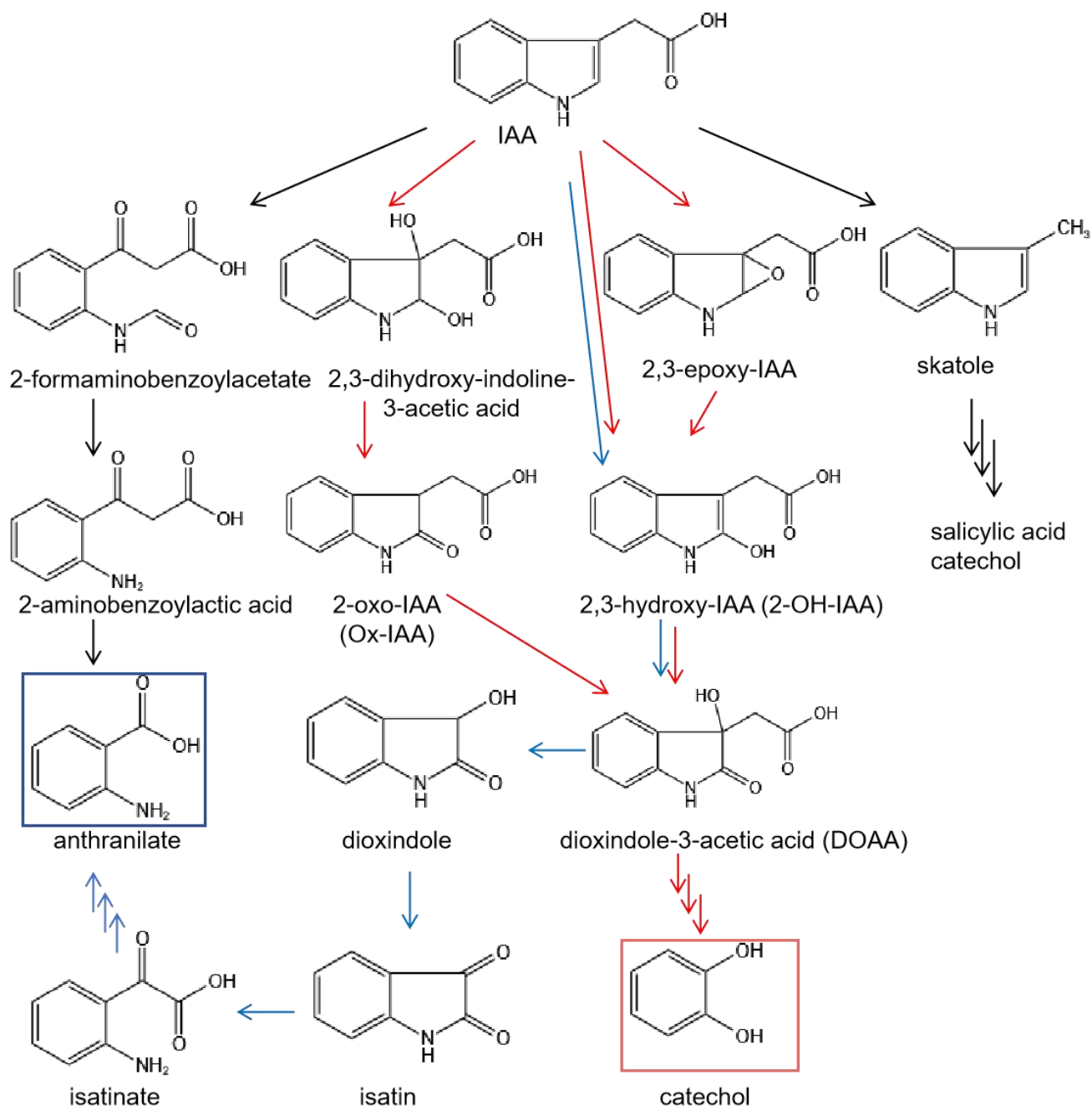

S1 Fig

Supplement: S1 Fig — Blue arrows showed the iad-pathway, the typical examples are Bradyrhizobium japonicum [23] and Variovorax paradoxus [24]. Red arrows showed the iac-pathway, the typical examples are Pseudomonas putida, Paraburkholderia phytofirmans, and Caballeronia glathei [18,20,22]. Black arrows showed pathways were not well classified so far. In 1961, Tsubokura and colleagues reported that IAA was biotransformed to 2-formaminobenzoylacetate and fruther decomposed into anthranilate by a bacterium isolated from air [70]. It is reported that Lysinibacillus xylanilyticus strain MA transformed tryptophan to IAA and then decarboxylated to produce 3-methylindole (skatole) [71], and the downstream pathways of skatole catabolism were firstly identified in Pseudomonas (Migula) in 1958 [72]. (PDF) [file pbio.3002921.s001.pdf]

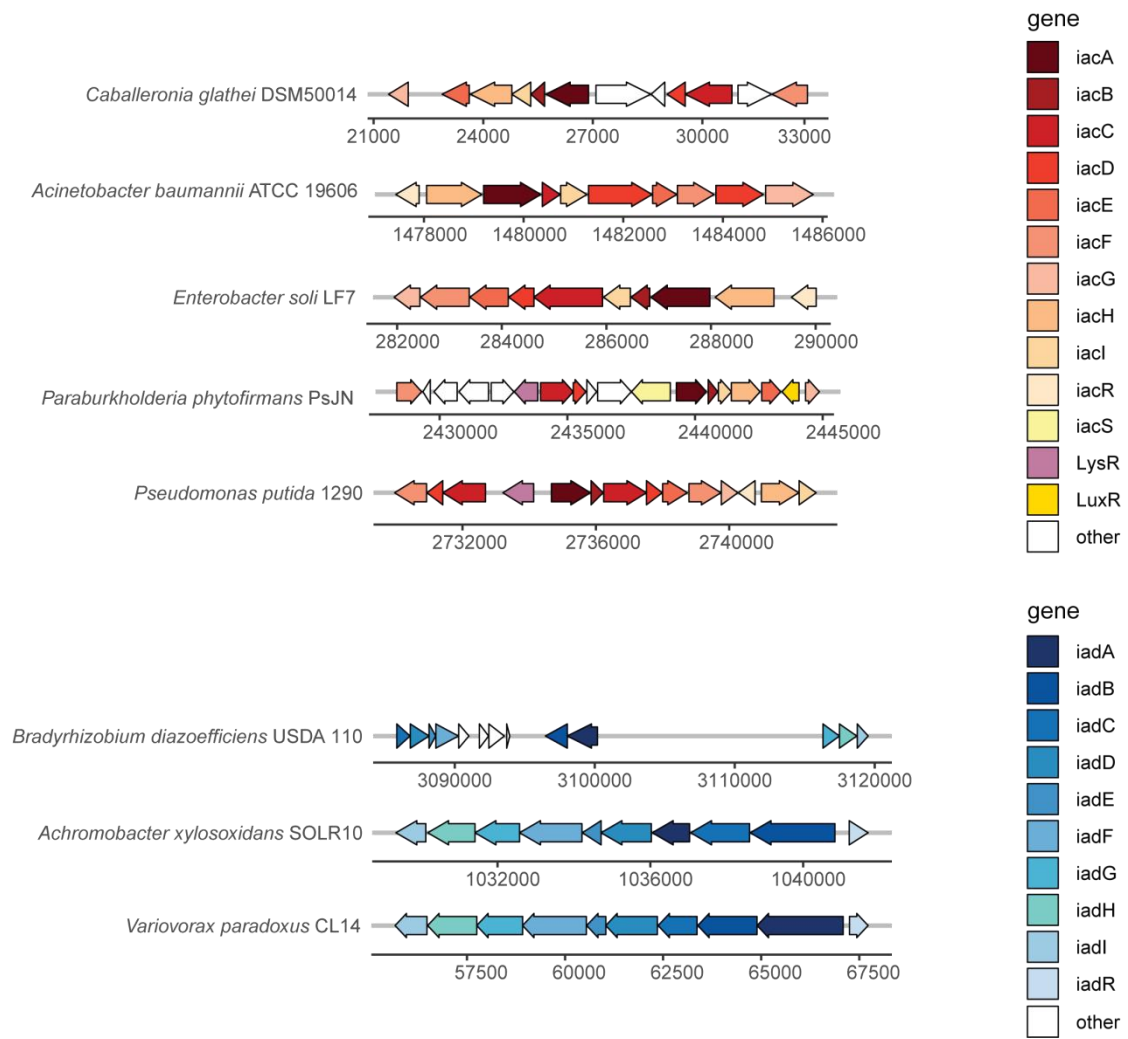

S2 Fig

Supplement: S2 Fig — (PDF) [file pbio.3002921.s002.pdf]

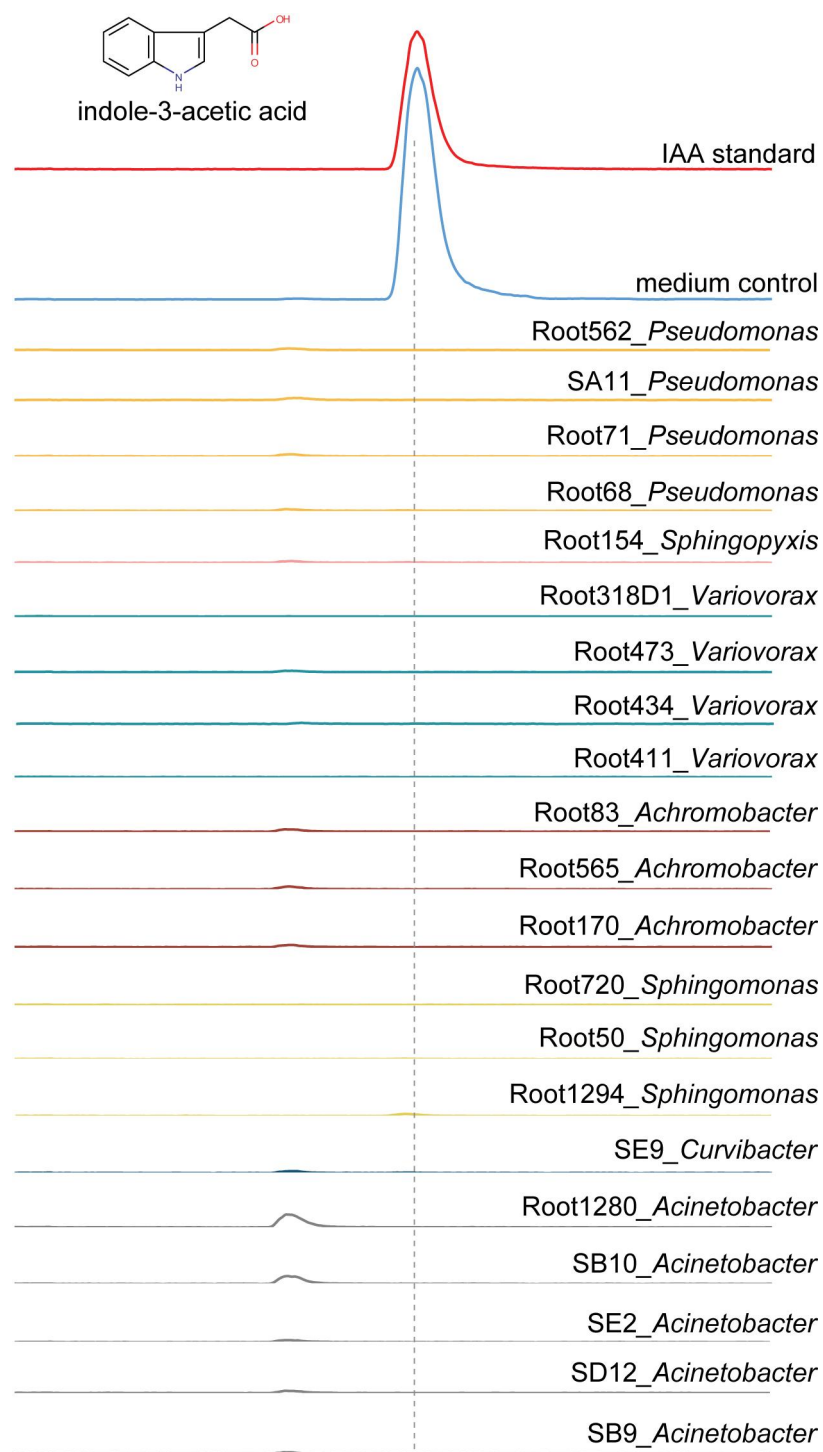

S4 Fig

Supplement: S4 Fig — Retention time of IAA in samples are consistent with commercial standard. (PDF) [file pbio.3002921.s004.pdf]

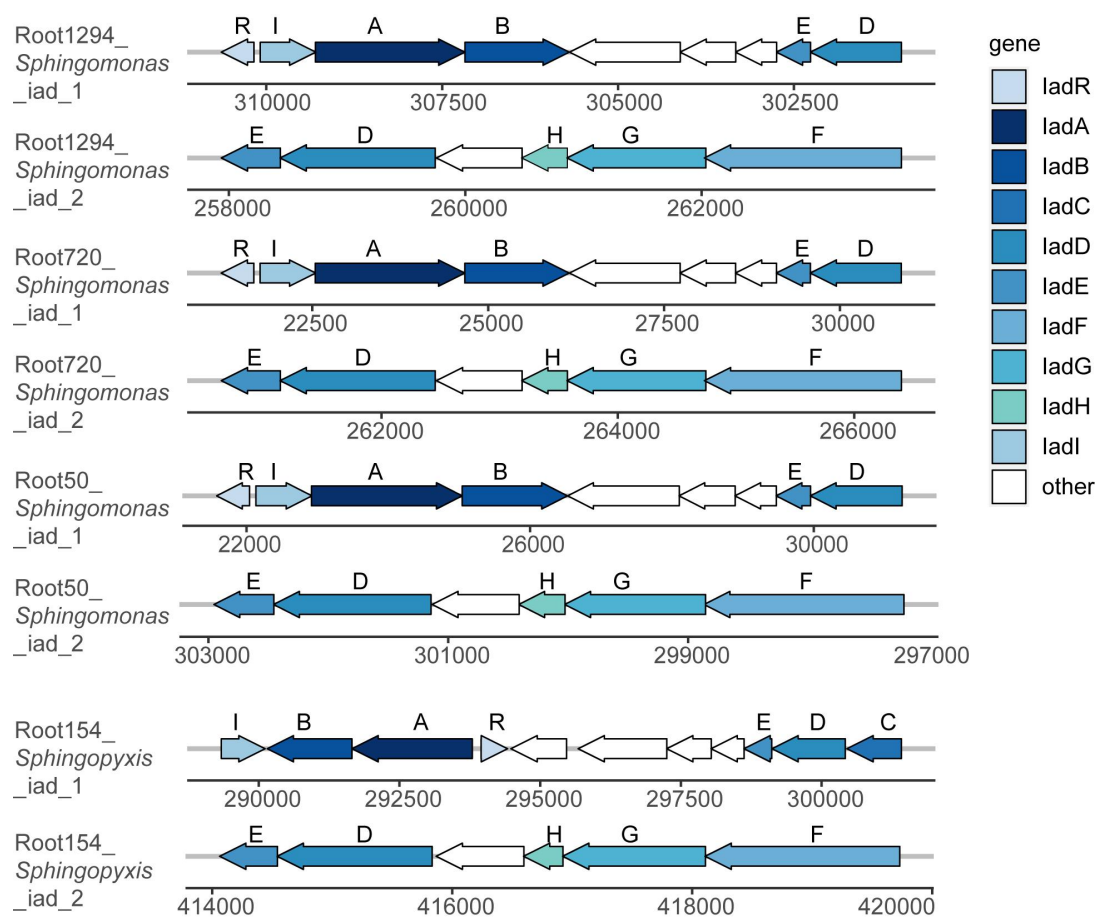

S5 Fig

Supplement: S5 Fig — (PDF) [file pbio.3002921.s005.pdf]

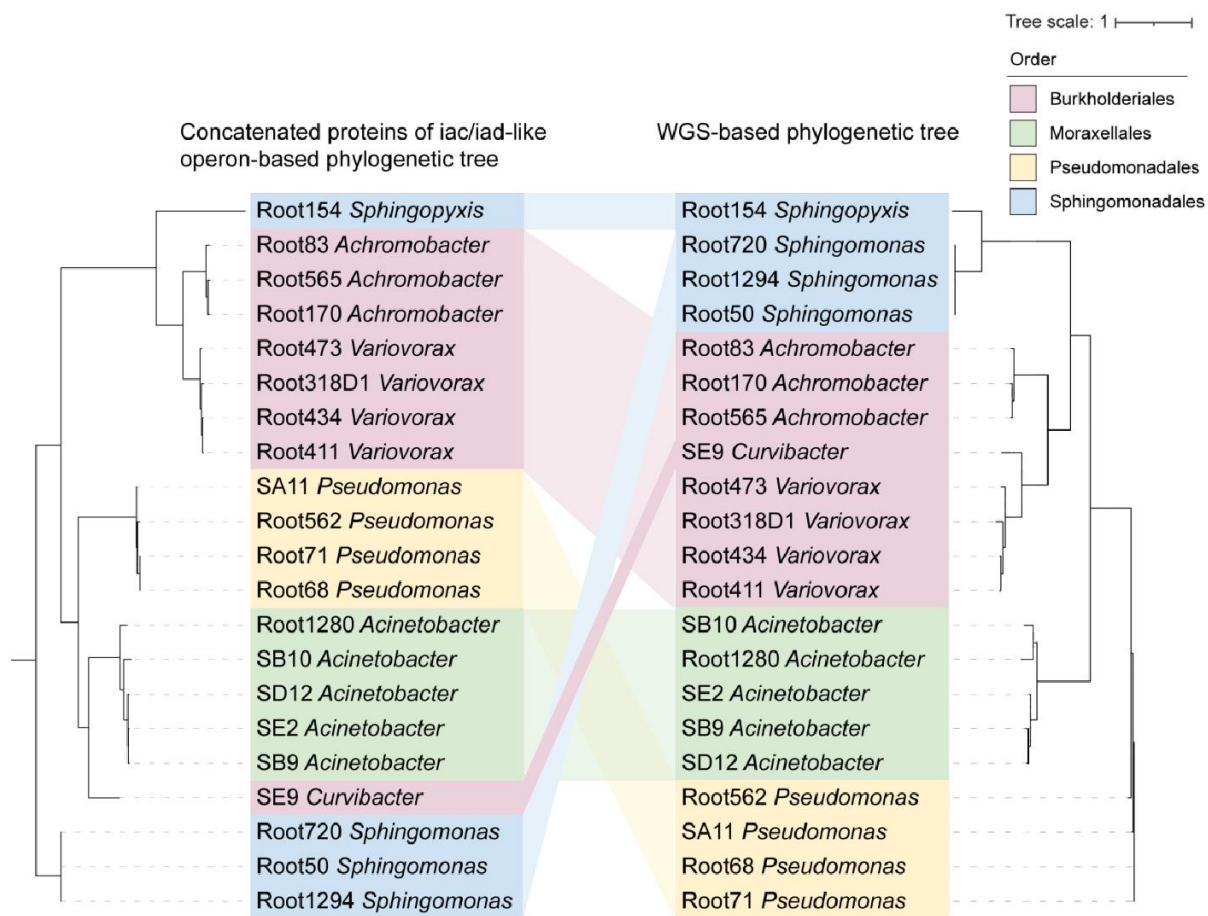

S6 Fig

Supplement: S6 Fig — (PDF) [file pbio.3002921.s006.pdf]

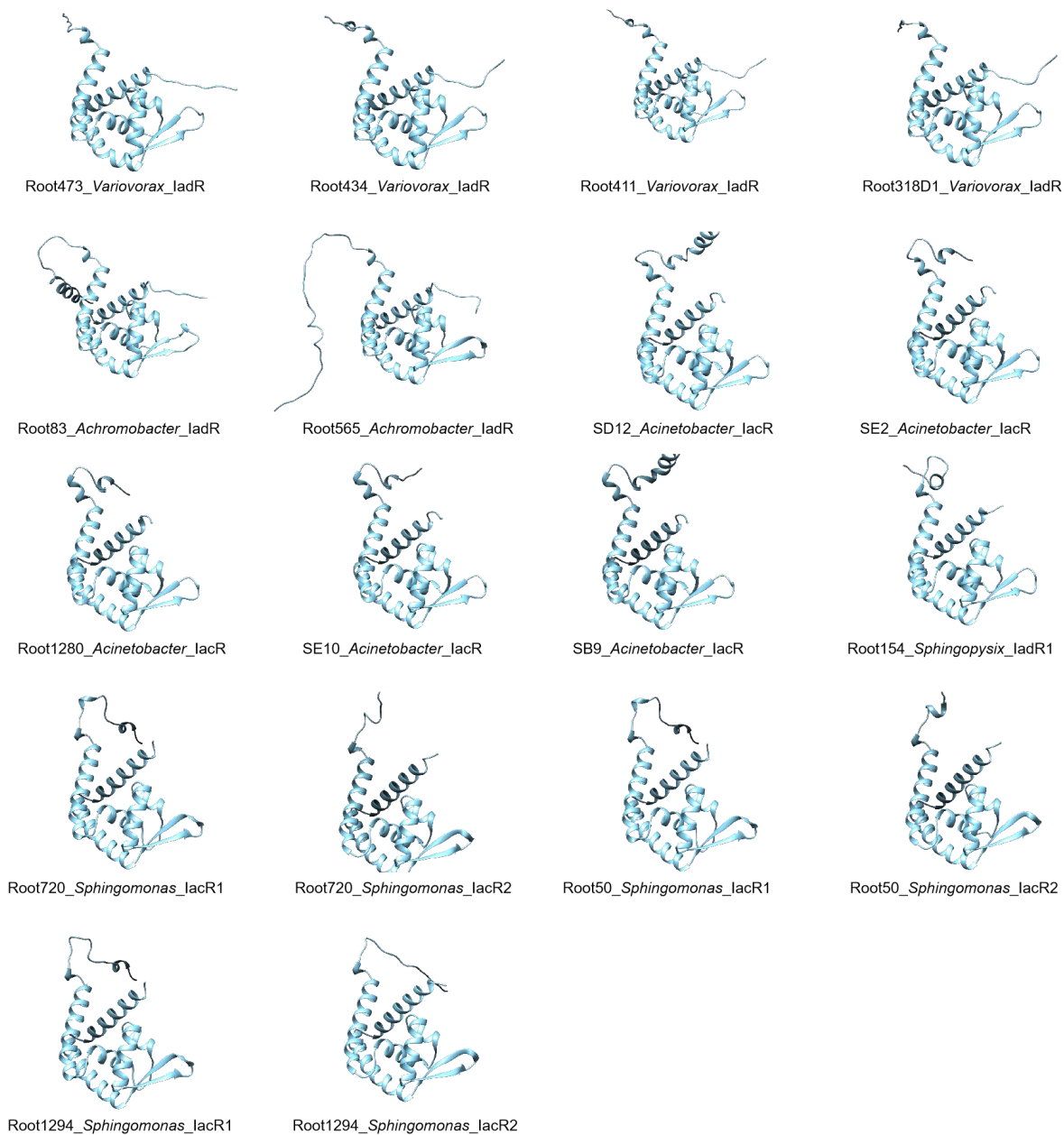

S8 Fig

Supplement: S8 Fig — All the structures were predicted by AlphaFold2. (PDF) [file pbio.3002921.s008.pdf]

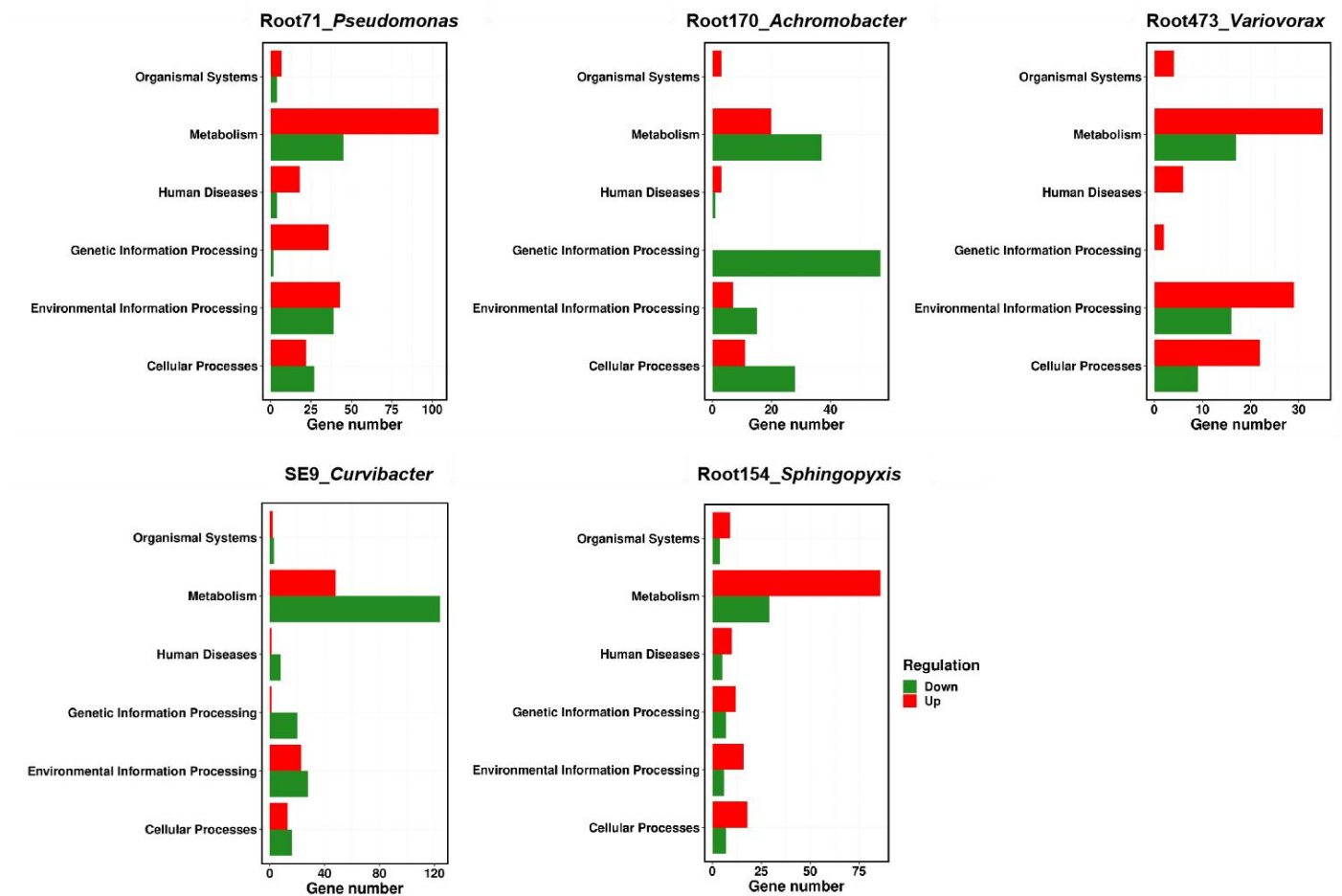

S9 Fig

Supplement: S9 Fig — Numerical values that underlie the data displayed in the panels are in S6 Table. (PDF) [file pbio.3002921.s009.pdf]

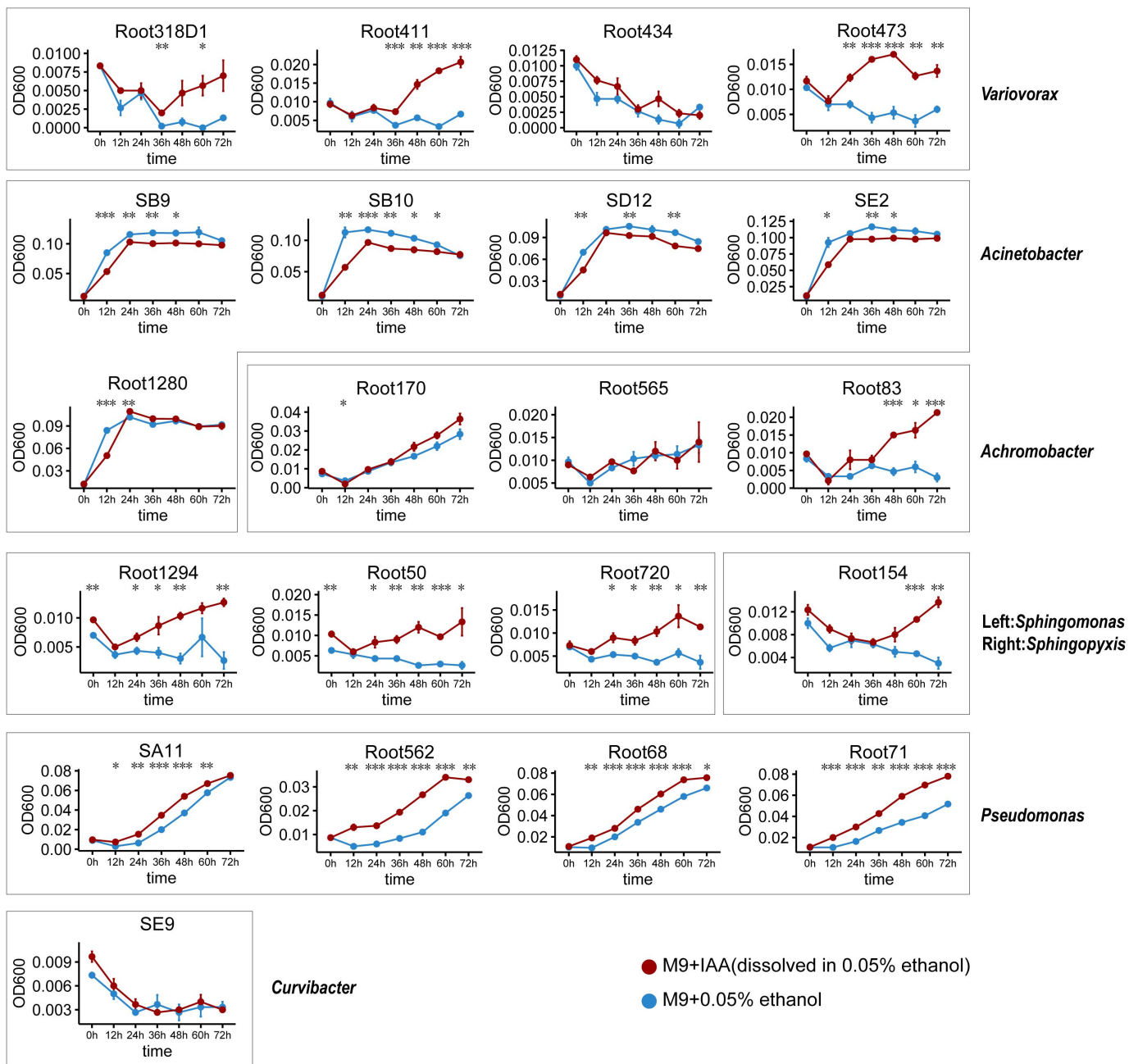

S10 Fig

Supplement: S10 Fig — Growth of individual IAA-degrading strains was measured at OD600 in M9 minimal medium supplemented with IAA (dissolved in ethanol) or M9 minimal medium supplemented with 0.05% ethanol as the sole carbon source (n = 3). Significant differences compared between M9+IAA and M9+0.05% ethanol were determined using Student’s t test: *P < 0.05, **P < 0.01, ***P < 0.001. Numerical values that underlie the data displayed in the growth curves are in S6 Table. (PDF) [file pbio.3002921.s010.pdf]

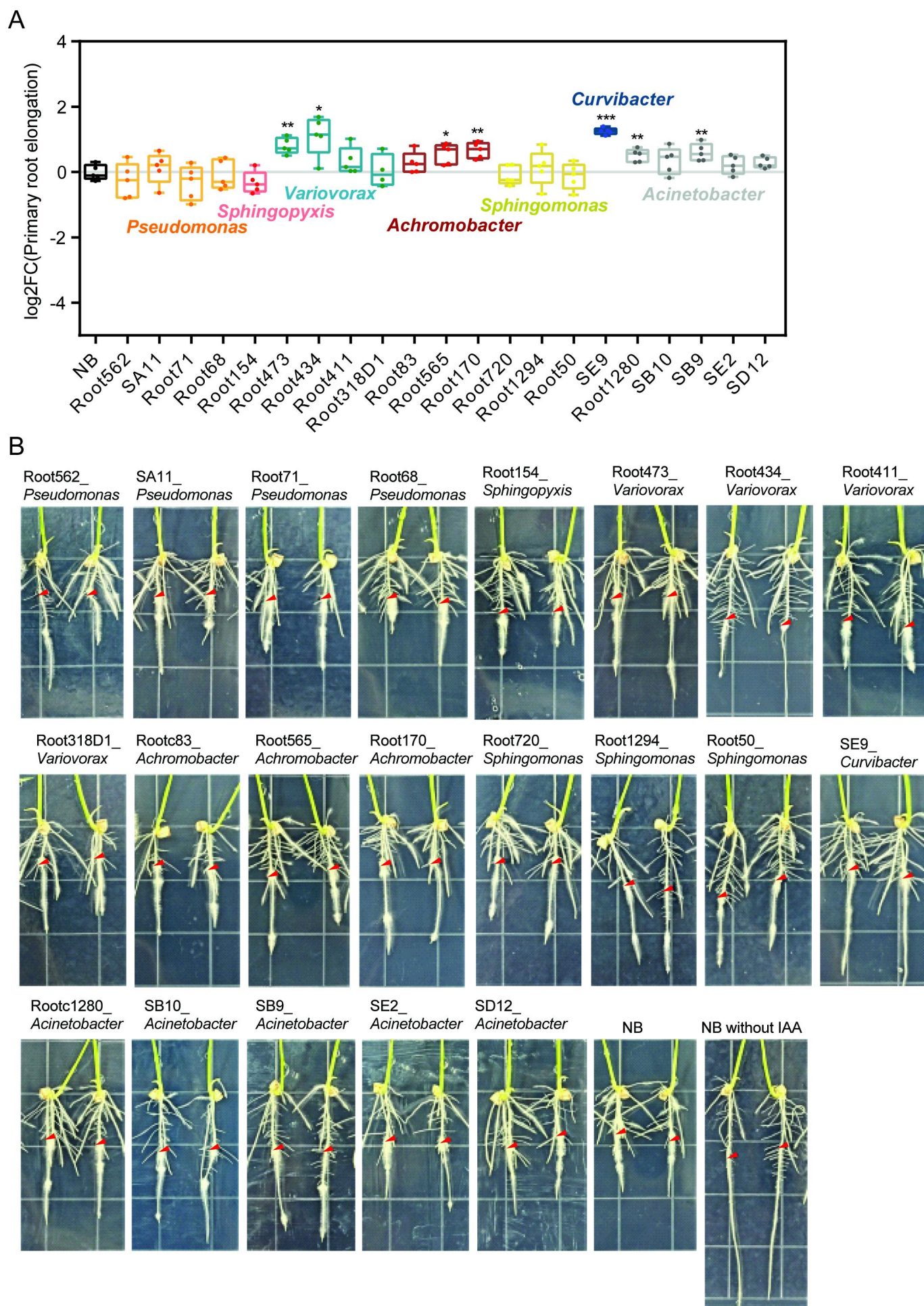

S11 Fig

Supplement: S11 Fig — (A) Exogenous IAA induced RGI was suppressed by some of the IAA-degrading strains. Boxplot middle line, median log2fold change of primary root elongation (vs. NB); box edges, 25th and 75th percentiles; whiskers, from the minimum to the maximum value. Each point represents a biological replicate, with 4 to 6 replicates were used in this experiment. (B) The images of rice seedlings grown on half strength MS agar plate supplemented with 10 μm IAA at 3 days after inoculation with or without bacteria. Red arrows indicate the original positions of the root tips at the time of bacterial inoculation. NB, No bacterial inoculation. Significant differences compared with NB group were determined using Student’s t test: *P < 0.05, **P < 0.01, ***P < 0.001. Numerical values that underlie the data displayed in the plot are in S6 Table. (PDF) [file pbio.3002921.s011.pdf]

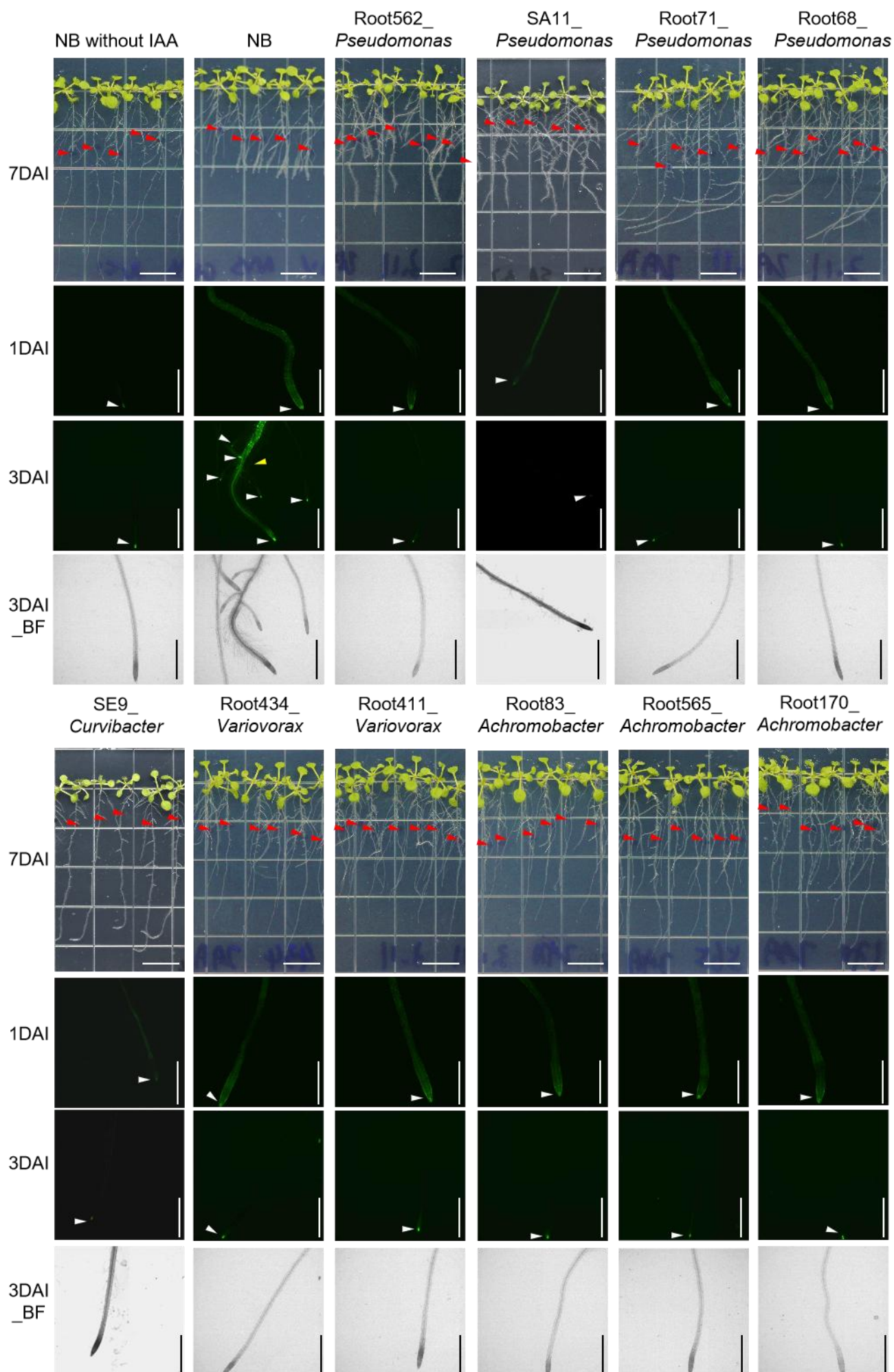

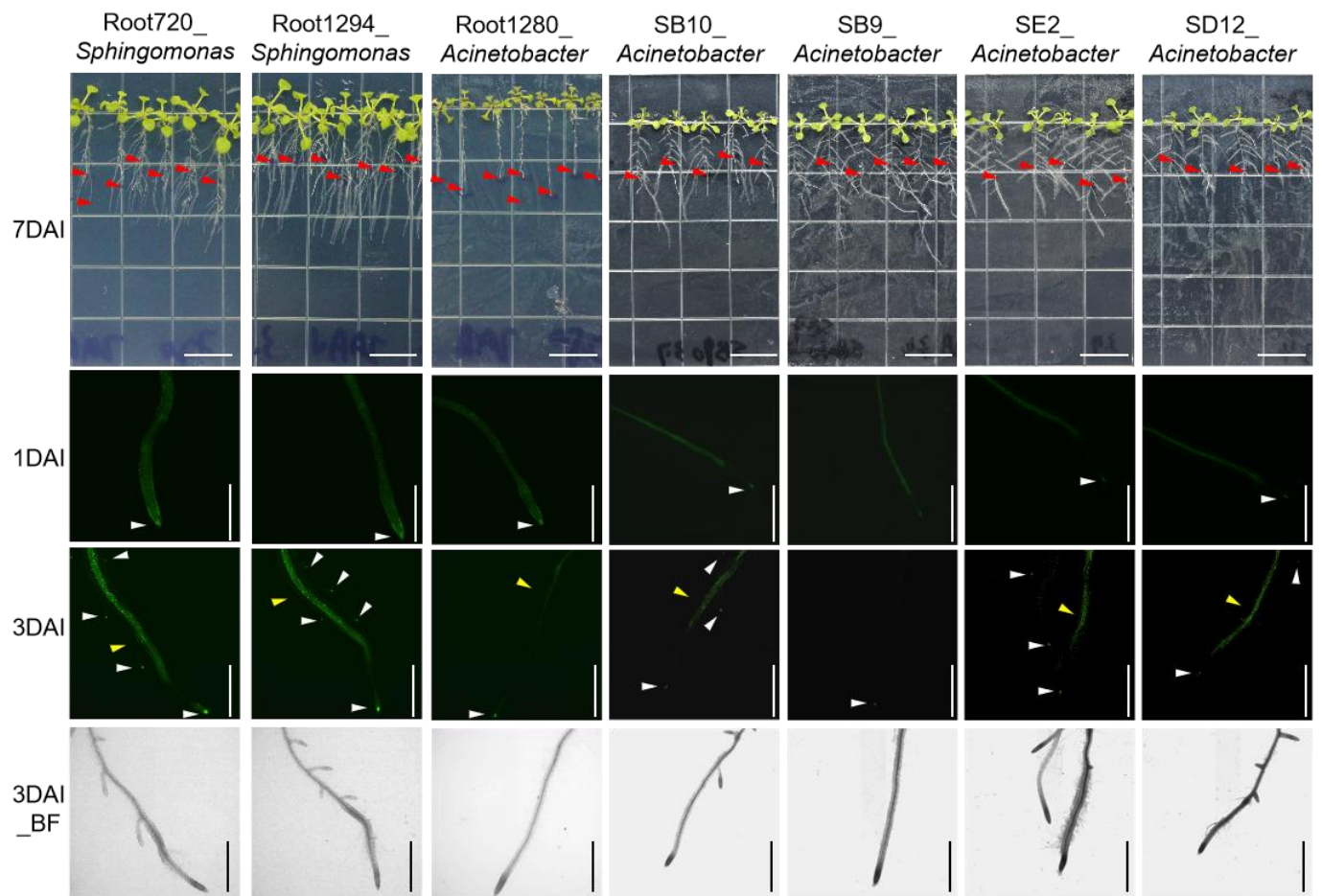

S12 Fig

Supplement: S12 Fig — The upper panels show the images of seedlings grown on 1/2 strength MS agar plate supplemented with 100 nM IAA, taken 7 days after inoculation with or without bacteria. Scale Bar = 1.4 cm. NB, No bacterial inoculation. Red arrows indicate the original positions of the root tips at the time of bacterial inoculation. The other panels show images of the primary roots of DR5::GFP plants following bacterial inoculation at 1 and 3 days postinoculation. Scale Bar = 1 mm. Write arrow shows the GFP signals on root tip. Yellow arrow shows the GFP signals on root which was induced by exogenous IAA. BF, bright field. (PDF) [file pbio.3002921.s012.pdf]

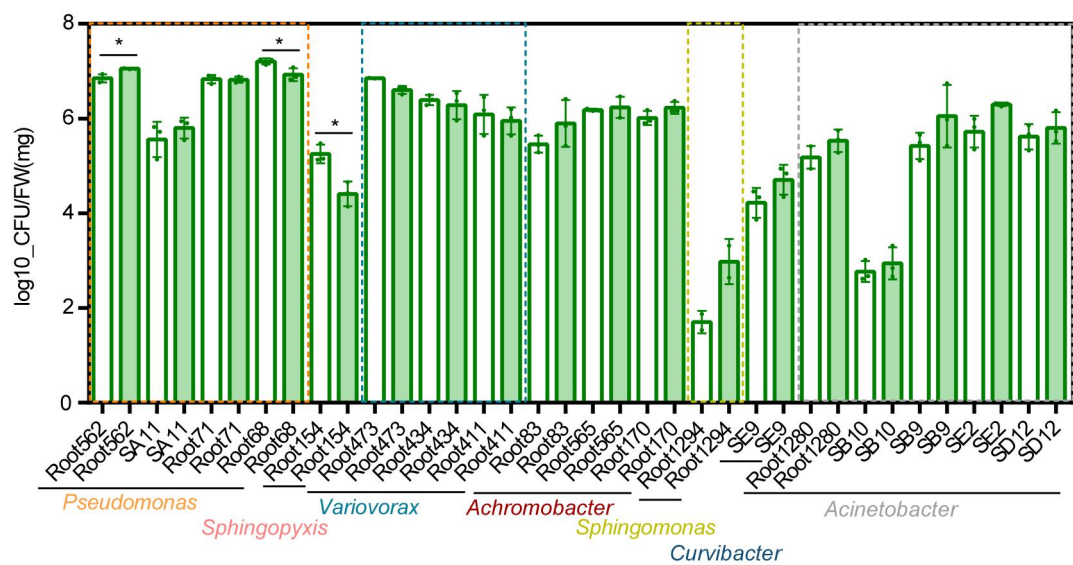

S13 Fig

Supplement: S13 Fig — The log-transformed CFUs of the IAA-degrading bacteria normalized to corresponding root weight on half-strength MS agar (open bar) and MS agar supplement with 100 nM IAA (solid bar) (n = 3). Numerical values that underlie the data displayed in the graph are in S6 Table. (PDF) [file pbio.3002921.s013.pdf]

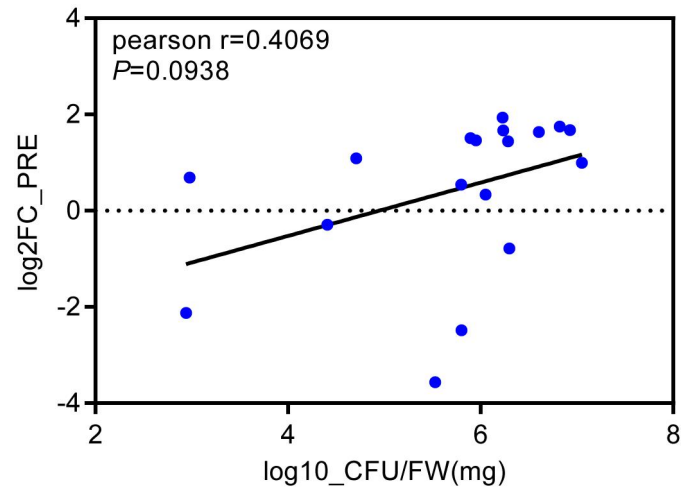

S14 Fig

Supplement: S14 Fig — Each blue dot shows mean of 3 to 4 biological replicates for 18 IAA degraders. Numerical values that underlie the data displayed in panel are in S6 Table. (PDF) [file pbio.3002921.s014.pdf]

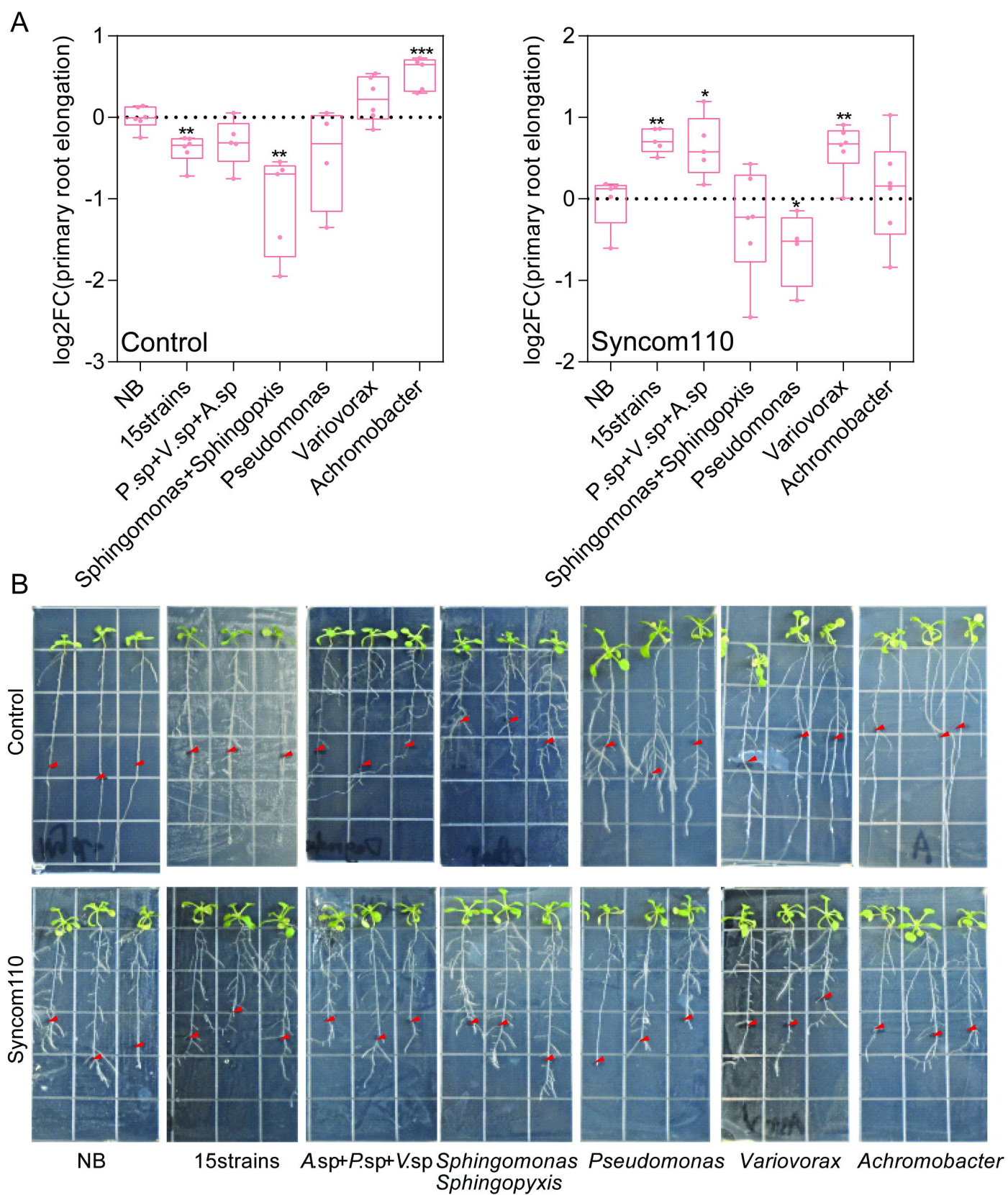

S15 Fig

Supplement: S15 Fig — (A) The complex synthetic bacterial community (comprising 110 bacterial strains, see S4 Table) induced RGI was partially alleviated by introducing IAA-degrading strains. Boxplot middle line, median primary root elongation; box edges, 25th and 75th percentiles; whiskers, from the minimum to the maximum value. Each point represents a biological replicate, with 4 to 6 replicates were used in this experiment. (B) The images of Arabidopsis seedlings grown on half-strength MS agar plates inoculated with or without bacteria at day 7. Red arrows indicate the original positions of the root tips at the time of bacterial inoculation. NB indicates no bacterial inoculation. Significant differences compared with NB group were determined using Student’s t test: *P < 0.05, **P < 0.01, ***P < 0.001. Numerical values that underlie the data displayed in the panels are in S6 Table. (PDF) [file pbio.3002921.s015.pdf]
