# Supplementary material for: Systematic characterization of plant-associated bacteria that can degrade indole-3-acetic acid
Source: PLoS Biol. 2024 Nov 26;22(11):e3002921. doi: 10.1371/journal.pbio.3002921 (PMC11630574; doi:10.1371/journal.pbio.3002921)
Supplement: S3 Fig — The whole genome sequence-based phylogenetic tree of the 183 strains was generated with phylophlan and visualized with iTOL. Genes annotated as iac-like or iad-like operons (with over 40% identity and 60% coverage compared to template amino acid sequences) were labeled with triangles. (PDF) [file pbio.3002921.s003.pdf]

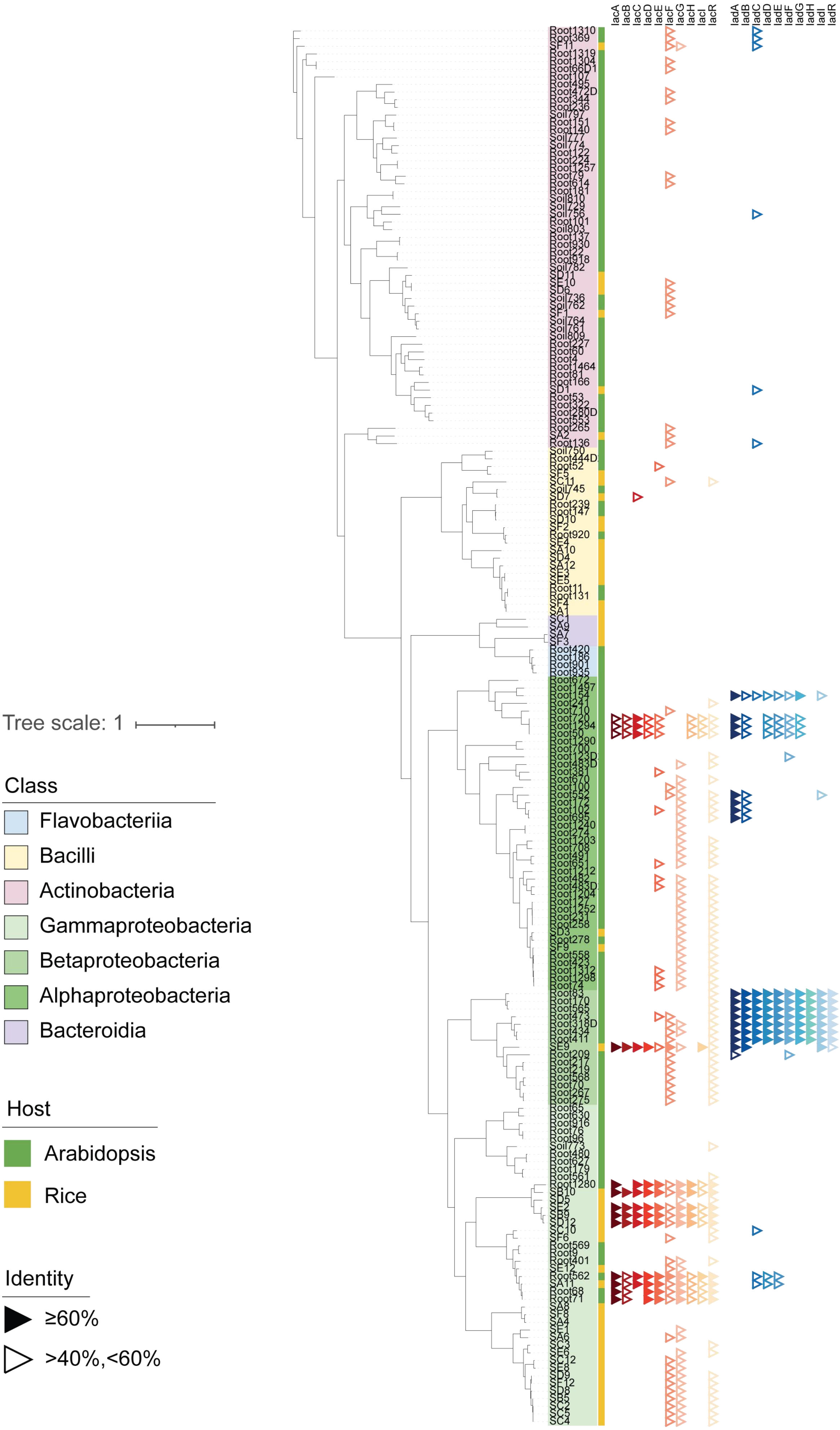

lacA
lacB
lacC
lacD
lacE
lacF
lacG
lacH
lacI
lacR
ladA
ladB
ladC
ladD
ladE
ladF
ladG
ladH
ladI
ladR
Tree scale: 1
Class
Flavobacteriia
Bacilli
Actinobacteria
Gammaproteobacteria
Betaproteobacteria
Alphaproteobacteria
Bacteroidia
Host
Arabidopsis
Rice
Identity
≥60%
>40%,<60%

S3 Fig
