# Supplementary material for: Systematic characterization of plant-associated bacteria that can degrade indole-3-acetic acid
Source: PLoS Biol. 2024 Nov 26;22(11):e3002921. doi: 10.1371/journal.pbio.3002921 (PMC11630574; doi:10.1371/journal.pbio.3002921)
Supplement: S7 Fig — Six MarR templates used in this analysis (labeled in red) are 7L1I Acinetobacter baumannii, 7KUA Pseudomonas putida, 7L19 Enterobacter soli ATCC BAA-2102, 7KYM Bradyrhizobium japonicum, 3CDH Ruegeria pomeroyi DSS-3, and 7KFO Variovorax paradoxus CL14. (PDF) [file pbio.3002921.s007.pdf]

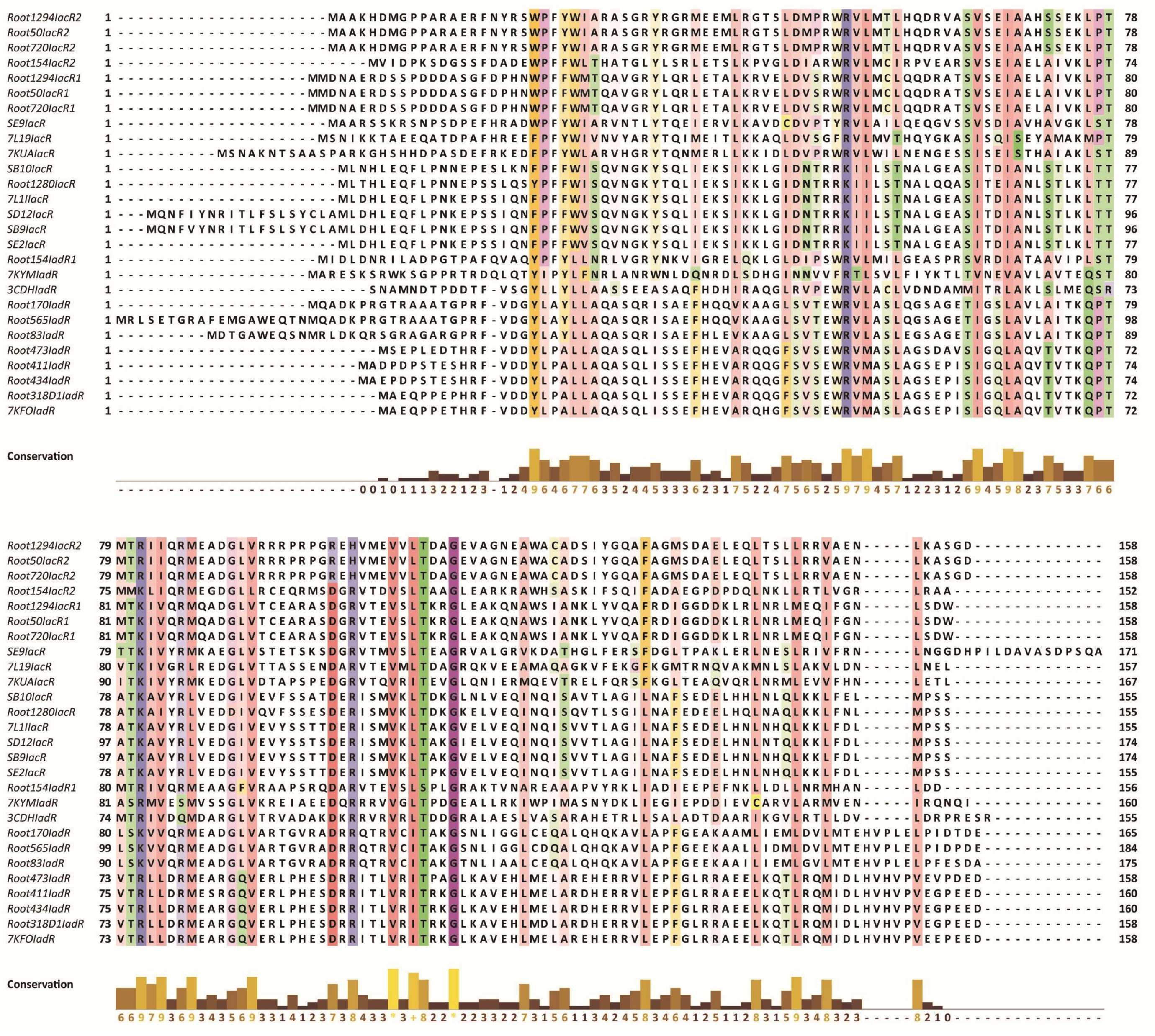
Root1294IacR2
Root50IacR2
Root720IacR2
Root154IacR2
Root1294IacR1
Root50IacR1
Root720IacR1
SE9IacR
7L19IacR
7KUAIacR
SB10IacR
Root1280IacR
7L1IIacR
SD12IacR
SB9IacR
SE2IacR
Root154IadR1
7KYMIadR
3CDHIadR
Root170IadR
Root565IadR
Root83IadR
Root473IadR
Root411IadR
Root434IadR
Root318D1IadR
7KFOIadR
Conservation
Conservation

S7 Fig
