# Supplementary material for: Systematic characterization of plant-associated bacteria that can degrade indole-3-acetic acid
Source: PLoS Biol. 2024 Nov 26;22(11):e3002921. doi: 10.1371/journal.pbio.3002921 (PMC11630574; doi:10.1371/journal.pbio.3002921)
Supplement: Attachment — Submitted filename: Response to Reviewers0708.pdf [file pbio.3002921.s022.pdf]

Response to Reviewer #1:

Point 1.0: This manuscript describes the analysis of bacterial isolates from Arabidopsis and rice roots. Genomic information was generated and used to identify genes that are known to be involved in the degradation of the plant hormone IAA. Twenty-one bacterial strains capable of degrading IAA were identified. Using transcriptomic analysis the authors show that in the presence of IAA the pathways involved I degrading IAA are upregulated in some bacteria and down regulated in others. A putative transcriptional regulator that controls expression of the IAA degrading pathway was identified. Additionally the authors evaluate metagenome assemblies and make observations regarding the presences of IAA degraders in different habitats.

Response: We greatly appreciate the valuable feedback provided by the reviewer on our work. Below we provide a point-by-point response to the reviewer's comments.

Comments:

Point 1.1: Title: the paper is focused on plant-associated bacteria. Consider changing title to reflect this.
Response: Thank you for your comment. We have revised the title as "**Degradation of indole-3-acetic acid by plant-associated bacteria**".

Abstract:
Point 1.2: Line 54: when referring to the regulator, since no studies were performed to confirm this role, this should be stated as "the putative regulator of the operon….."
Response: We have revised the sentence according to your suggestion. "**Additionally, the putative regulator of the operon was found to be highly conserved among these strains through protein structure similarity analysis.** "

Introduction:
Point 1.3: Line 107: iadR, should be written as IadR if referring to the protein. Also authors should clarify if this is a MarR homolog that they are referring to, to aid the reader and give context.
Response: Thank you for your comment, we have revised the sentence as "**For example, the crystal structure and binding properties of IadR, a member of the MarR family, were recently determined in *V. paradoxus*, revealing the mechanism of operon expression regulation**".

Point 1.4: Line 114: "presence of iacAE and iadDE". Should this be "or" vs "and".
Response: We have revised it.

Point 1.5: Line 115: Readers will not know what "Salkowski method" is. Authors should use another term or remove from introduction.
Response: Thank you for your suggestion, we have removed this term.

Point 1.6: Line 122: except, instead of expect?
Response: Thank you for your comment, we have revised it.

Results:
Point 1.7: Lines 142-150: Strains do not exhibit sequence identity. Please clarify that you are referring to the predicted proteins in the genomes of these strains.
Response: Thank you for your comment, we have revised it. Please see line 137-147 in page 6. "**Among these, predicted proteins encoded by the genomes of four *Pseudomonas* strains, five *Acinetobacter* strains, and one *Curvibacter* strain show high amino acid sequence identity to the reported IacA and IacE (identity > 60%). Additionally, predicted proteins encoded by the genomes of four strains belonging to *Variovorax* and three to *Achromobacter*, exhibit high amino acid sequence identity to the experimentally validated IadD and IadE. A strain belonging to *Sphingopyxis* harbors genes encoding predicted proteins display moderate amino acid sequence identity to the IadD and IadE (identity between 40% to 60%). Interestingly, the genomes of three *Sphingomonas* strains contain both *iacA/E* and *iadD/E*, which encode the proteins with moderate amino acid sequence identity to the templates (Figure 1) (Supplementary Table2).**"

Point 1.8: Lines 149: What constitutes "medium" amino acid identity?
Response: We have replaced "medium" with "moderate" to avoid any misunderstanding.

Point 1.9: It is not clear how the authors are choosing isolates for follow up, There are initially 189 bacterial isolates total (137 from Arabidopsis and 52 from rice). Authors identify 21 that have the genes they are looking for. But then when they go to validate predicted results, they evaluate 137 Arabidopsis isolates and only 6 candidates from the rice collection. Please explain. The authors identify 32 out of 143 strains that are able to degrade IAA (>30%), but focus on 21 strains for further follow up. I may have missed it but it wasn't clear to me why the focus was placed solely on the 21 strains that degraded >50% of IAA? Did the authors investigate the other 11 strains further? Presumably they contain proteins predicted to be IacAE or IadDE?
Response: Thank you for your comments, we have conducted additional experiments to validate all the bacterial strains studied in this manuscript. Also, we have included the data of the IAA metabolism capabilities of all strains in new **Figure1**. We removed 6 isolates from Arabidopsis root-associated collections due to the lack of whole genome sequences. Currently, this study includes 183 isolates: 131 from Arabidopsis root-associated collections, and 52 from rice root-associated collections. We further focused on the 11 strains which exhibited weak IAA degradation capabilities. None of these strains contain

proteins predicted to be members of iac or iad-like operon (please see **Figure 1** and **Supplementary Figure 3**). The degradation efficiency of these strains was tested, and compared to the beginning of the experiment, the IAA content in the medium was reduced by less than 30% (please see **Figure1**).

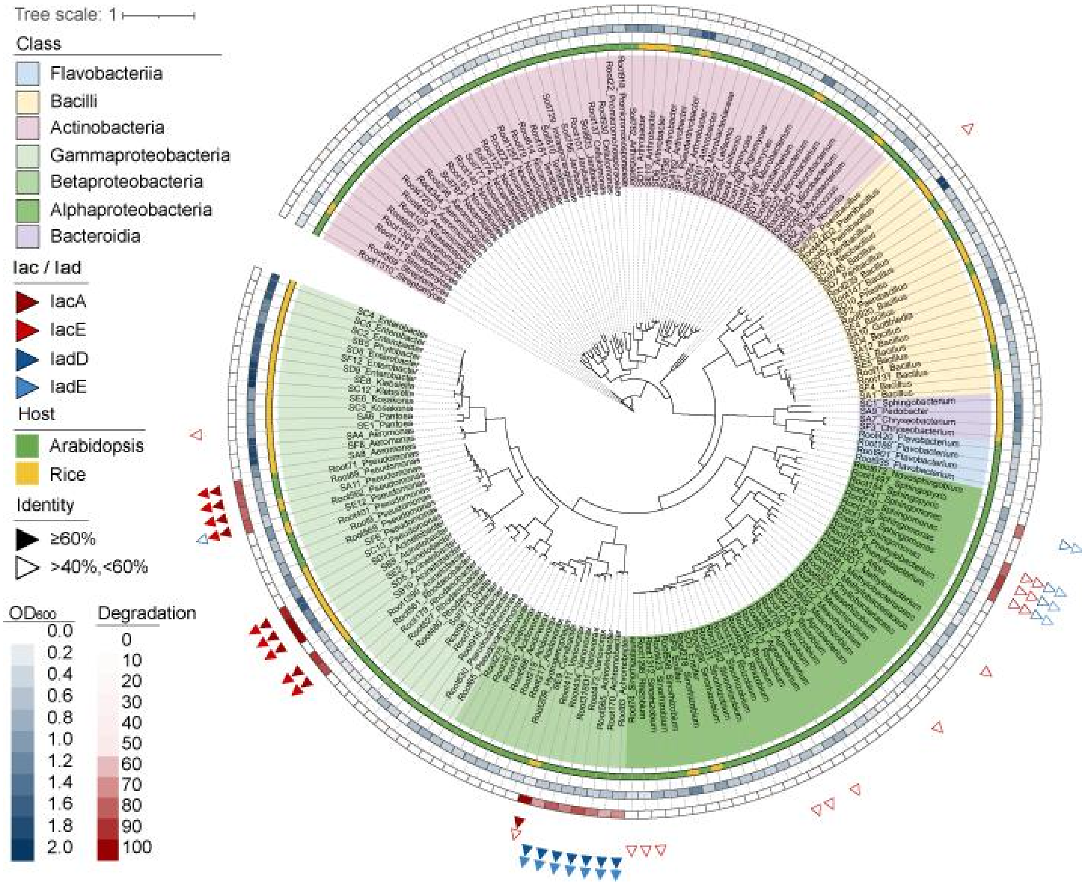

Figure 1. Bacterial strains isolated from Arabidopsis and rice rhizosphere are annotated for genes related to IAA degradation. The whole genome sequence-based phylogenetic tree of the 183 strains was generated with phylophlan and visualized with iTOL. Strains annotated to have IacA/E or IadD/E (over 40% identity and 60% coverage with template amino acid sequences, see Methods) were labeled with triangles. The inner ring with blue colour represent bacterial biomass as measured by OD_{600} after 72 hours' growth in $^{1}/_{2}$ TSB. The outer ring with red colour represent the percentage of IAA degradation of individual strains after 72 hours' incubation in $^{1}/_{2}$ TSB medium performed by Salkowski method.

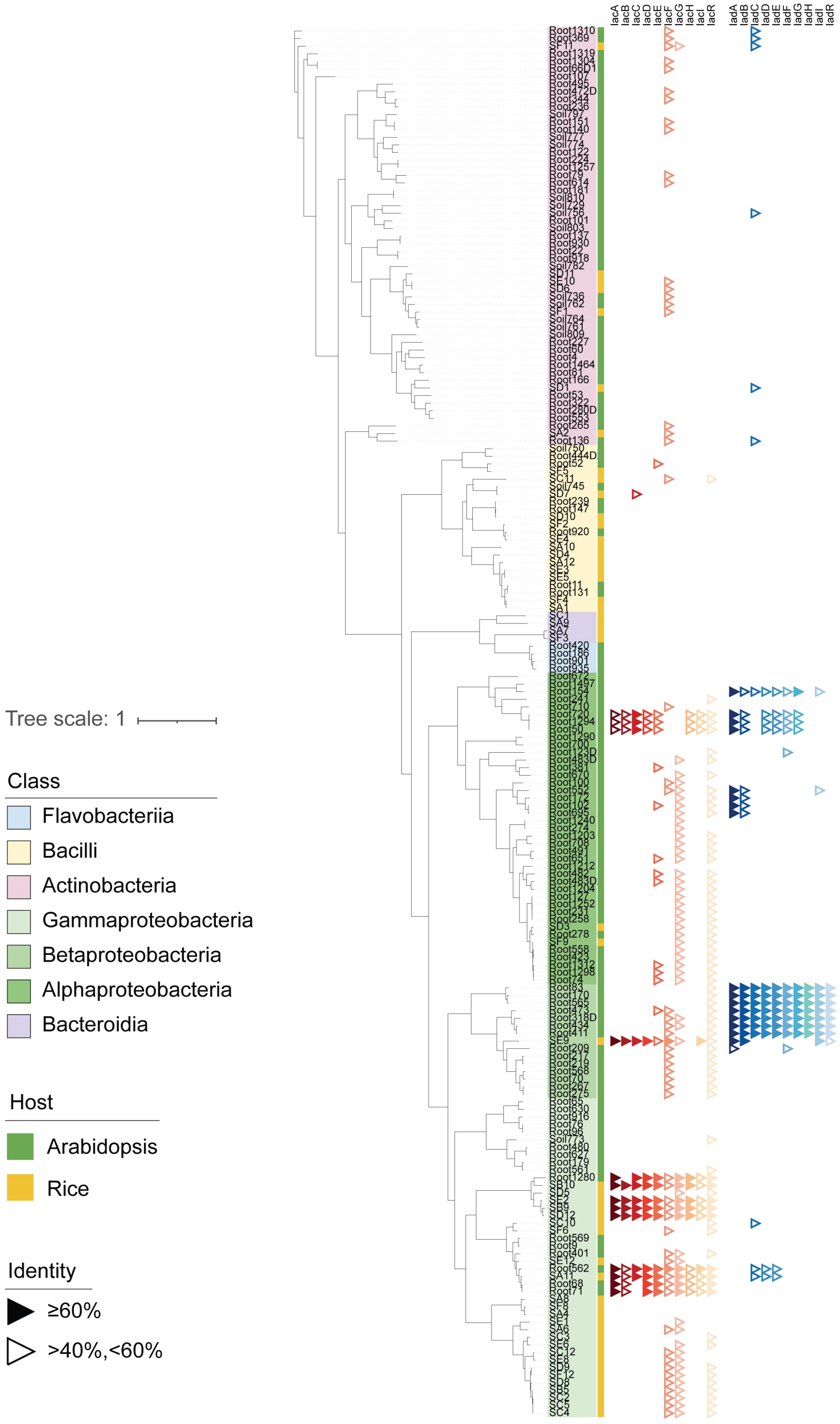

lacA
lacB
lacC
lacD
lacE
lacF
lacG
lacH
lacI
lacR
ladA
ladB
ladC
ladD
ladE
ladF
ladG
ladH
ladI
ladR
Tree scale: 1
Class
Flavobacteriia
Bacilli
Actinobacteria
Gammaproteobacteria
Betaproteobacteria
Alphaproteobacteria
Bacteroidia
Host
Arabidopsis
Rice
Identity
≥60%
>40%,<60%

Supplementary Figure 3. The IAA catabolism gene clusters were annotated in the bacterial strains. The whole genome sequence-based phylogenetic tree of the 183 strains was generated with phylophlan and visualized with iTOL. Genes annotated as iac-like or iad-like operons (with over 40% identity and 60% coverage compared to template amino acid sequences) were labeled with triangles.

Point 1.10: The authors construct a phylogeny tree based on 16sRNA and trees using the predicted IacAE or IadDE from the bacteria. If the authors found operons for these degradation pathways, it might be more useful to use all of the genes in the operons rather than limit the phylogeny to a few of the proteins. Also, since the authors have the whole genomes, did the authors look at phylogeny based on whole genome (vs 16s rRNA)? What do authors mean by "results suggest that evolution of microbes un-synchronized with some gene cluster acquisition"?

Response: Thank you for your comments, we have rebuild the phylogeny tree using the whole genome sequences of the strains in new **Figure 1**, **Supplementary Figure 3**, and **Supplementary Figure 6**, and rebuild the phylogeny tree using the concatenated iac or iad gene cluster encoded amino acid sequences in **Figure 2A** and **Supplementary Figure 6**.

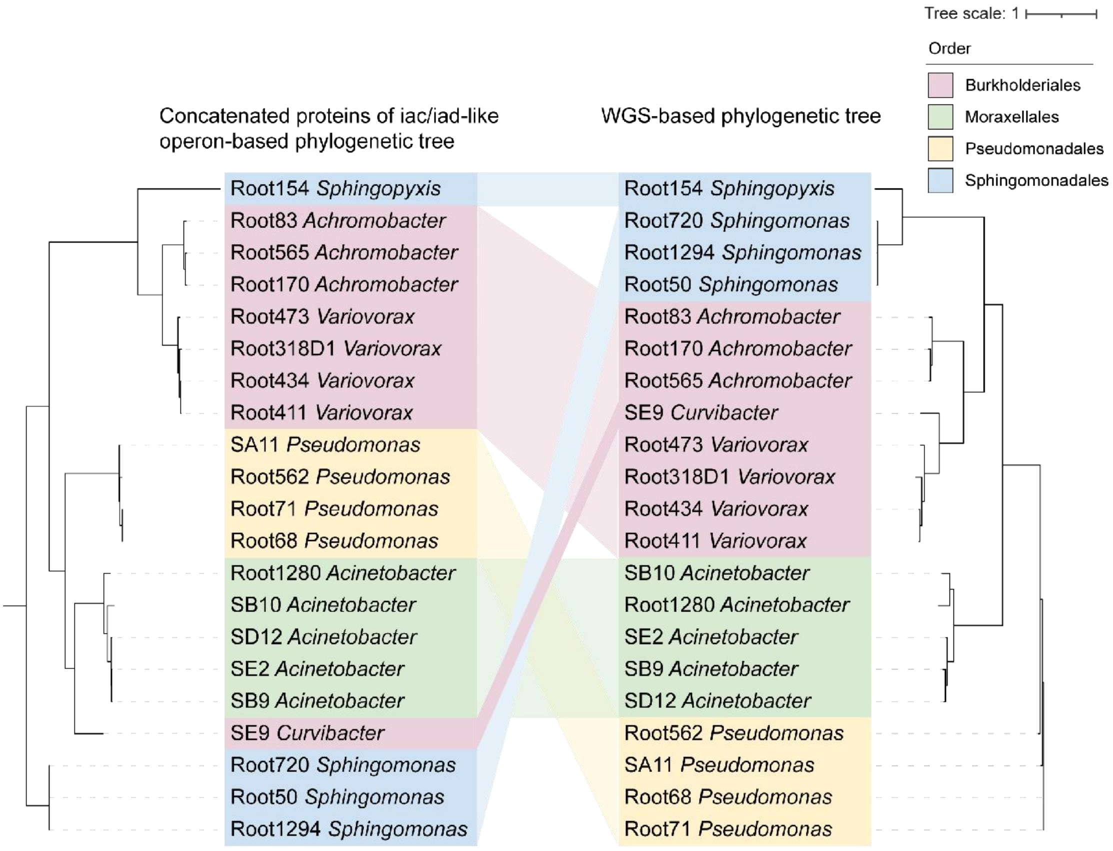

Supplementary Figure 6. The phylogenetic tree based on concatenated IacA and IacE or IadD and IadE amino acid sequences vs. the phylogenetic tree based on 16S rRNA.

"results suggest that evolution of microbes un-synchronized with some gene cluster acquisition". As shown in Supplementary Figure 6, there is no one-to-one correspondence between phylogenetic tree based on the WGS of strains and the sequence of complete operon. We have revised this sentence to avoid any misunderstanding. Please see line 191-193 in page 7. “**These findings suggest that the evolutionary processes of these strains do not align perfectly with the acquisition of certain gene clusters. This discrepancy implies that the evolution of the microbes and the integration of these specific gene clusters into their genomes occurred at different rates or times, indicating a lack of synchronization between these two processes.”**

Point 1.11: Line 246: authors should indicate the genus for "Root154"
Response: Thank you for your comment. We have revised it, and we have indicated the genus for “RootXXX” throughout the manuscript.

Point 1.12: Line 150-261: authors should indicate the genus for all places that have "RootXXX"
Response: Thank you for your comments, we have revised it throughout the manuscript.

Point 1.13: Line 279-281: Authors do not have any evidence of the presence of a by product.
Response: Thank you for your comment. Since we have no evidence of the presence of byproducts, we have removed the inappropriate wording from this sentence.

Point 1.14: Line 288: Do authors mean ½ strength MS agar?
Response: Yes, we have revised “1/2” to “**half-strength**” or “$^{1}/_{2}$“ throughout the manuscript to avoid any misunderstandings.

Point 1.15: Line 321: The authors use iacA/E and iadD/E to search genomes of bacteria from various habitats. Is there a reason why they did not use the full operons for the search? The authors refer to the operons later on, but as written seems like they only used two genes to search the genomes.
Response: Thank you for your comment and we understand your concerns. There are experimental evidence show that IacA and IacE of the iac operon, and IadD and IadE of the iad operon are essential for IAA bio-transformation in the representative strains *C. glathei* and *V. paradoxus*, respectively. Therefore, we used these proteins as biomarkers to search our 183 strains in this study, resulting in the selection of 21 target strains (**Figure1**). We also search the genomes of the 183 strains for the complete operon, and identified the same strains (**Supplementary Figure 3**). As experimentally validated, only these 21 screened strains have the ability to catabolize IAA (**Figure1**). Thus, we used the biomarker proteins as templates to search the MAG database. When hits were found, they were identified as containing the operon.

Discussion:

Point 1.16: Lines 365: Do authors have evidence about the habitats for IAA producers? Are producers found in other habitats that degraders are not?

Response: Thank you for your comment. In this sentence, “IAA producer” means plant and microbes which have the ability to produce IAA. To avoid any misunderstanding, we revised this sentence as “**Intriguingly, MAG analysis shows that IAA-degrading candidates prefer to colonize plant-associated habitats, which seems to suggest that IAA degraders naturally coexist with IAA producers (here mainly referring to plants and IAA-synthesizing microbes)**”. Please see line 383-386 in page 13.

If IAA producers refer specifically to microbes, there is currently no comprehensive study on their habitats to our knowledge. However, there are many independent research articles report that microbes isolated from soil, rhizosphere, phyllosphere, or endophytes can produce IAA. In fact, one of the typical characteristics of many PGPR (plant growth promoting rhizobacteria) is their ability of IAA production [1]. Importantly, research has shown that gut microbiota and airway microbe can also synthesize IAA and this compound is closely related to human health [2, 3]. Conducting a systematic investigation into the distribution of IAA producers would be a valuable and meaningful study, but it is not the focus of this research. Regarding the overlapping habitats of IAA producers and IAA degraders, more data needs to be collected.

Point 1.17: Lines 423-428: This paragraph can be omitted.

Response: We have deleted the paragraph according to your suggestion.

Methods:

Point 1.18: Lines 501: Do authors mean ½ strength. This needs to be corrected throughout the manuscript.

Response: Thank you for your comments, we have revised “1/2” to “**half-strength**” or “**$^{1}/_{2}$**“ throughout the manuscript to avoid any misunderstandings.

Point 1.19: For the RNA-Seq studies, it is not clear how the comparisons were made. What is IAA dissolved in? Did the authors include a control for this? How did the authors map to a reference genome? These genomes are draft genomes. Perhaps so readers are not confused authors should indicate the reads were mapped to the corresponding draft genomes?

Response: Firstly, we apologize for not explaining our experimental details clearly, which caused the confusion raised by the reviewer. We dissolved IAA in ethanol. The control group (glucose treatment) supplemented the same volume of ethanol to exclude any effects from the solvent. We have revised the methods section to provide more details. Please see line 564-567 in page 18. “**Root71_*Pseudomonas*, Root170_*Achromobacter*, Root473_*Variovorax*, SE9_*Curvibacter*, and Root154_*Sphingopyxis* were grown in M9 medium supplemented with 1 mM IAA or 1.712 mM glucose (same volume of ethanol was added in glucose treatments)**. ”

You are right; these genomes are draft genomes and we mapped the reads to the corresponding draft genomes. To avoid any misunderstandings, we have provided more details in the methods section according to your suggestions. Please see line 582-583 in page 19. “**The clean reads were then mapped to the draft genome of corresponding strain with STAR (v2.7.10a)**”.

Point 1.20: Line 568: Please clarify 2-3 days until cloudy.
Response: We have revised the sentence to avoid any misunderstanding. Please see line 590-591 in page 19. “**Strains selected for *in planta* assays were pre-cultured in half-strength TSB at 25°C, 200 rpm for 2 to 3 days**.” Based on our experience, after 2 to 3 days pre-culture, the OD_{600} of the bacterial culture could reach above 0.5.

Point 1.21: Line 601: Pleas clarify "flipped sideways".
Response: Thank you for you comment, we have added details in this section to avoid any misunderstandings. Please see line 648-652 in page 21. “**A 5 uL liquid sample was placed on the far left side of the ½ TSB agar plate. The plate was slowly tilted to the right to allow the liquid to flow evenly. Once the liquid reached a certain point, the plate was slowly tilted to the left, causing the remaining liquid to move back towards the left side**”.

Point 1.22: Line 876: "deferential" is mis-spelled
Response: Thank you for your comments, we have revised it.

Point 1.23: Figure 4: It is very difficult to see the percentage of degradation and growth of strains in (A) and (B) because so many are plotted on the graph. For (E), Why evaluate 7 days in top panel, but 3 days for other panels. These should be evaluated on the same day, unless there is a good reason? Also for the legend significant differences are determined by comparison to a control group, however on the graphs there are 2 controls, "IAA control" and "MS control". Please clarify.
Response: Thank you for your comments. For panel A and B, we have changed the Y-axis to separate the plots. Please see the new **Figure 4**. For panel E, the phenotype of seedlings on day 7 after inoculation is more significant. Also we measured the primary root elongation at day 7 after inoculation, so the phenotype images here are from 7 days post-inoculation.

For the GFP image, we found the IAA induced fluorescence is quenched after inoculated with IAA-degrading bacteria (Root473_*Variovorax*) at day 3 post-inoculation, thus we stopped imaging.

“MS control” refers to seedlings grown on half-strength MS agar without bacterial inoculation. Since this control treatment is not necessary and may cause misunderstanding, we have removed the “MS control” in this figure. Please see the new **Figure 4**. We also renamed “IAA control” as “NB”, which means no bacterial inoculation

on the seedlings grown on half-strength MS agar supplemented with IAA. All treatment were compared with the NB control.

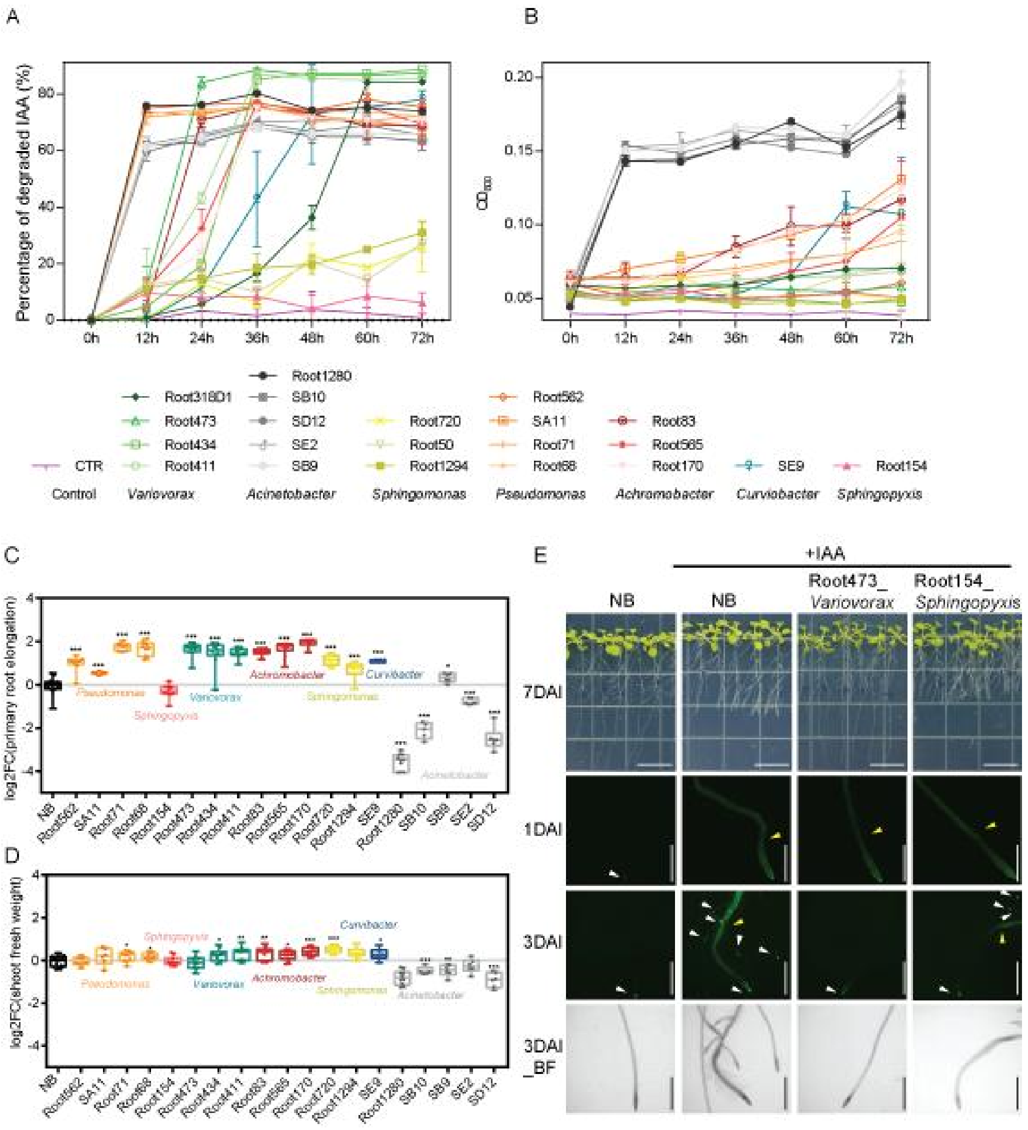

Figure 4. IAA-degrading strains can utilize IAA and suppress the IAA-induced root growth inhibition. (A), IAA consumed by IAA degraders *in vitro* and (B) their growth as measured by OD_{600} in M9 minimal medium supplemented with IAA as the sole carbon source (n=4). (C), Except for strains from genus of *Sphingopyxis* and *Acinetobacter*, IAA-induced root growth inhibition was suppressed by IAA-degrading strains. Boxplot middle line, median $\log_2$ fold change of primary root elongation (vs NB); NB, No bacteria inoculation. box edges, 25th and 75th percentiles; whiskers, 1.5× the interquartile range. Left to right n = 16, 10, 6, 10, 10, 10, 10, 10, 10, 10, 10, 10, 10, 10, 6, 10, 5, 6, 6, and 8 biological replicates. (D), Apart from *Acinetobacter*, strains inoculation have no negative effects on shoot fresh weight. Boxplot middle line, median $\log_2$ fold change of shoot fresh weight (vs NB); box edges, 25th and 75th percentiles; whiskers, 1.5× the interquartile range. Left to right n = 17, 10, 10, 6, 10, 10, 10, 10, 10, 10, 10, 10, 10, 10, 10, 10, 7, 9, 9, and 19 biological replicates. (E), Images of representative Col-0 seedlings grown axenically (NB) or with

IAA-degrading strain inoculation. Upper panes show the representative images of seedlings grown on half strength MS agar plate supplemented with 100nM IAA at 7 days after inoculation with or without strain. Bar = 1.4cm. The other planes show the representative primary root images of *DR5::GFP* plants after inoculated with strain for 1 and 3 days. Bar = 1mm. Write arrows show the GFP signal on root tips. Yellow arrows show the GFP signals on root which were induced by exogenous IAA. BF, bright field. Significant differences compared with control group were determined using Student's *t*-test: **P*<0.05, ***P*<0.01, ****P*<0.001.

Response to Reviewer #2:

Point 2.0: The plant hormone auxin (IAA) is important for many developmental processes in the plant. Some microbes can also produce IAA, however, the percentage of IAA-degrading or -consuming microbes in the rhizosphere has not been assessed in detail. In their article, Wang et al. investigate this using comparative genomics, metabolite analyses, in planta assays, and microbial transcriptomics.

The authors screened the genomes of 189 bacterial isolates originally cultured from Arabidopsis thaliana or rice roots for the presence of iac (IAA-catabolizing) or iad (IAA-degrading) pathways, and validate their findings with in vitro IAA degradation assays with those strains. They identify 21 strains, among which two bacterial genera have not yet been reported for IAA consumption/degradation before. The presence of the gene regulator of the MarR (multiple antibiotic resistance regulator) family proteins was also confirmed. Bacterial transcriptome analysis of five strains upon IAA-treatment revealed a strong regulation of genes related to the KEGG ontology "metabolism". Plants treated with IAA show root growth inhibition (RGI), and some but not all of the IAA-degrading strains can restore normal root growth when inoculated onto the Arabidopsis plants. Finally, a large number of bacterial metagenome assembled genomes that are available on public databases were screened for the presence of iad and iac genes to reveal that more potential degraders are present in terrestrial vs. aquatic environments.

The manuscript is structured in a clear way, the figures are intuitive and appealing. There are a few typos, and the English language should be thoroughly proofread.
Response: We greatly appreciate the valuable feedback provided by the reviewer on our work. Below we provide a point-by-point response to the reviewer's comments.

Main comments:
Point 2.1: While the performed experiments are clear and well-analyzed, the text remains a bit descriptive. This reviewer was missing a perspective on how the newly gained knowledge can be applied and how researchers can build up on it. The authors claim that they reveal "a key role" (l. 366) of IAA degraders, but it should be laid out more specifically

what this role is for host plants, because the in planta experiments are not convincing enough.

For example, how would these degraders perform within a rhizosphere community and is this biologically meaningful? This could be tested in the lab experiments by adding not only one but multiple strains to the agar-based assays.

Response: Thank you for your comments. According to your suggestion, we built a synthetic bacterial community by mixing 110 strains isolated from Arabidopsis rhizosphere. Inoculation with this synthetic community inhibit the primary root elongation in Arabidopsis seedlings, while RGI induced by this community inoculation was partial restored by IAA-degrading strains. We have added this part in result. Please see line 329-338 in page 11. "**To further investigate the biological roles of IAA degraders in a natural context, RGI induced by exogenous IAA treatment was replaced with a synthetic bacterial community formed from 110 Arabidopsis root isolates (hereafter referred to as Syncom110). Fifteen strains of IAA-degrading bacteria isolated from Arabidopsis roots were grouped into different clusters to assess their ability to counteract RGI induced by Syncom110 (Supplementary Table 4). The primary root elongation was severely inhibited by inoculation with Symcom110. Interestingly, only clusters containing *Variovorax* strains were able to partially revert the RGI caused by Syncom110 (Supplementary Figure 14). These results are consistent with previous report**".

Please see **Supplementary Figure 14**. We believe this part of results demonstrates the biological significance of the presence of IAA-degrading strains in natural scenario.

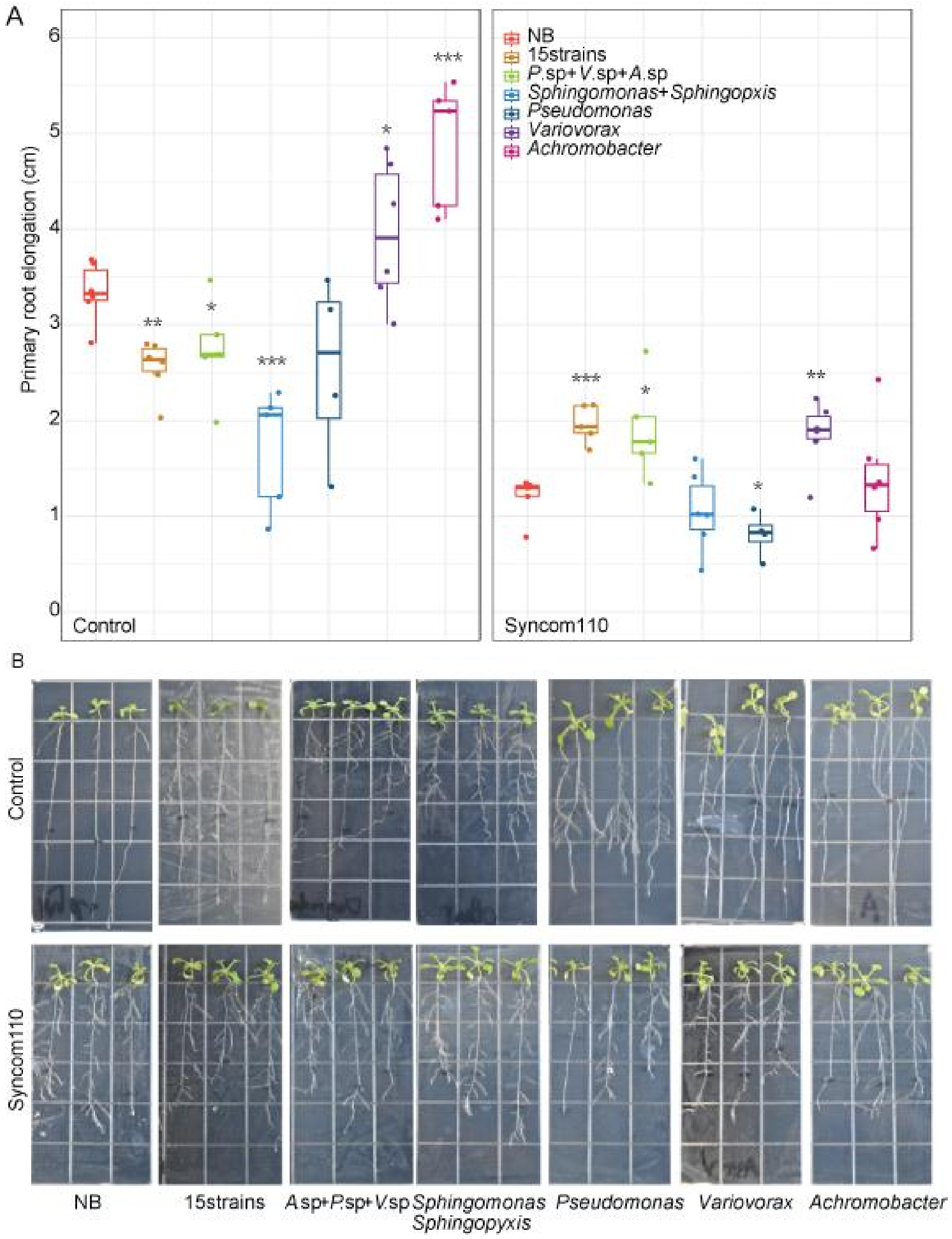

Supplementary Figure 14. IAA-degrading bacterial strains restore RGI caused by Syncom110 in Arabidopsis seedlings. (A), The complex synthetic bacterial community (comprising 110 bacterial strains, see Supplementary table 4) induced RGI was partially alleviated by introducing IAA-degrading strains. Boxplot middle line, median primary root elongation; box edges, 25th and 75th percentiles; whiskers, 1.5 × the interquartile range. Each point represents a biological replicate, with 4 to 6 replicates were used in this experiment. (B), The images of Arabidopsis seedlings grown on half-strength MS agar plates inoculated with or without bacteria at day 7. NB indicates no bacterial inoculation. Significant differences compared with NB group were determined using Student's *t*-test: **P*<0.05, ***P*<0.01, ****P*<0.001.

Point 2.2: All experiments are done in Arabidopsis, and how do the bacterial isolates affect rice root growth?

Response: Thank you for your comments. We supplemented our study with experiments on rice root growth inoculated with IAA-degrading strains. We found that excess IAA induced RGI partial restored by inoculating with strains of *Variovorax*, *Achrombacter*, *Curvibacter*, and *Acinetobacter*. The results of the rice inoculation differ slightly from those of Arabidopsis, which may be due to the different sensitivities of the two species to IAA. Please see line 306-309 in page 11. " **Additionally, in rice mono-associations, compared with axenic control, primary root elongation was observed when seedlings were inoculated with strains from *Variovorax*, *Achromobacter*, *Curvibacter* and *Acinetobacter* (Supplementary Figure 10).** "

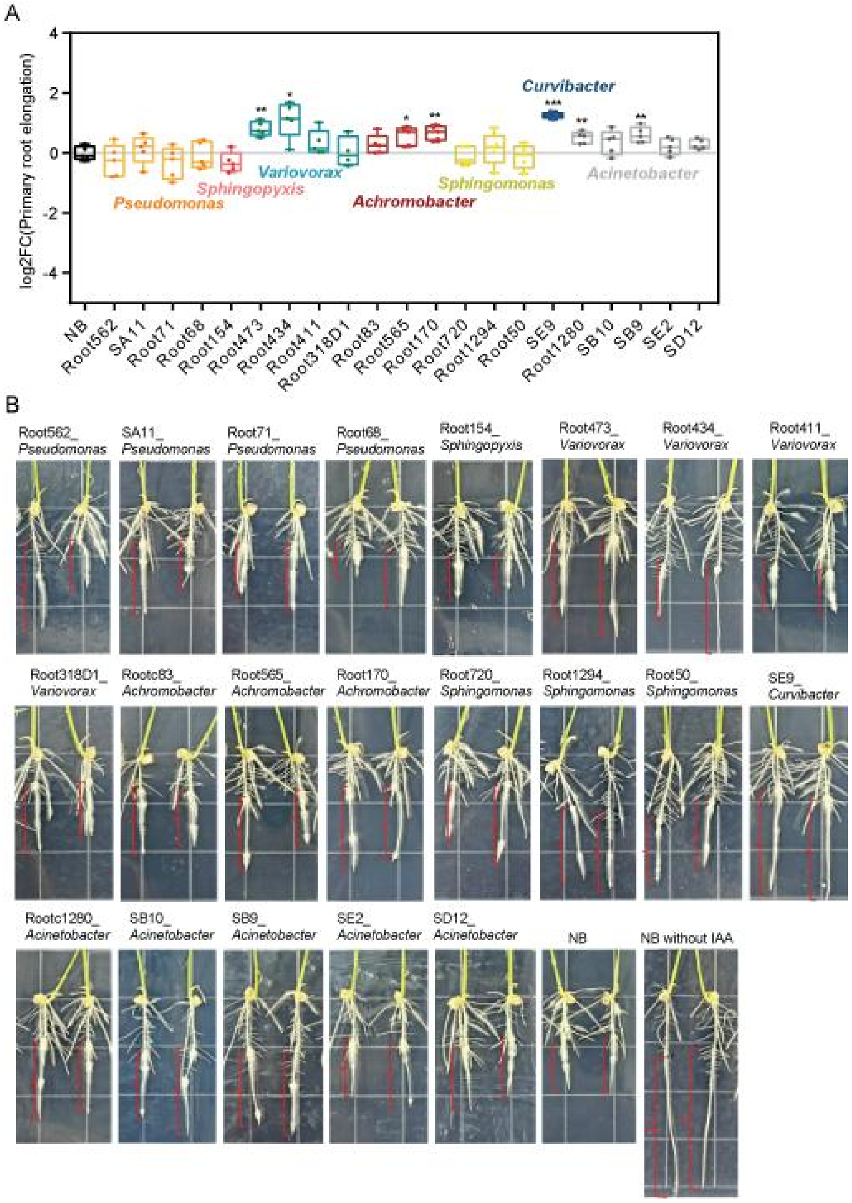

Supplementary Figure 10. IAA-degrading bacterial strains restore the RGI by exogenous IAA in rice seedlings. (A), Exogenous IAA induced RGI was suppressed by some of the IAA-degrading strains. Boxplot middle line, median $\log_2$ fold change of primary root elongation (vs NB); box edges, 25th and 75th percentiles; whiskers, 1.5 × the interquartile range.

Each point represents a biological replicate, with 4 to 6 replicates were used in this experiment. (B), The images of rice seedlings grown on half strength MS agar plate supplemented with 10μM IAA at 3 days after inoculation with or without bacteria. NB, No bacterial inoculation. Significant differences compared with control group were determined using Student's *t*-test: *P<0.05, **P<0.01, ***P<0.001.

Point 2.3: How do the authors explain that there is no DR5 induction by the RGI non-suppressive degraders (l. 309 ff)? If these strains degrade IAA in vitro but not in planta, what is the reason for it?

Response: It is possible that the RGI non-suppressive strains degrade IAA very slowly *in planta*, or that their growth and/or gene expression *in planta* differs from that observed *in vitro*. As a result, the IAA concentration in the $^{1}/_{2}$ MS agar remains at a level that inhibits Arabidospsis root growth. The relatively high concentration of IAA in the $^{1}/_{2}$ MS agar continuously activates the auxin reporter, so the GFP florescence remains present in the *DR5::GFP* plants inoculated with these strains.

Point 2.4: In the transcriptomic analysis, would IAA as sole carbon source affect the response? Wouldn't a more stringent control have been to use glucose in low concentrations and then plus/minus IAA? As shown in the growth assays, some strains grow fast and to high densities with IAA only, and this enhanced proliferation itself may affect bacterial transcriptional response.

Response: Thank you for your comment. We agree with your point, and thus we have supplemented our study with additional experiments. We cultured the strain in M9 medium supplemented with 1% glucose and 1mM IAA (dissolved in ethanol) as the treatment group, and M9 medium supplemented with 1% glucose and ethanol (same volume with IAA) as the control group. Two strains (Root71_*Pseudomonas* and Root473_*Variovorax*) were selected to assess their transcriptional responses under these different culture conditions. Because this study focuses on whether the iac or iad-like operon is regulated by IAA, we analyzed transcriptome data from two experiments, and found that in both cases, IAA up-regulated the operon. The slight differences observed in the results between the two experiments may be due to variations in the medium and sampling times, but they do not affect our conclusion that the iac or iad-like operon is up-regulated by IAA.

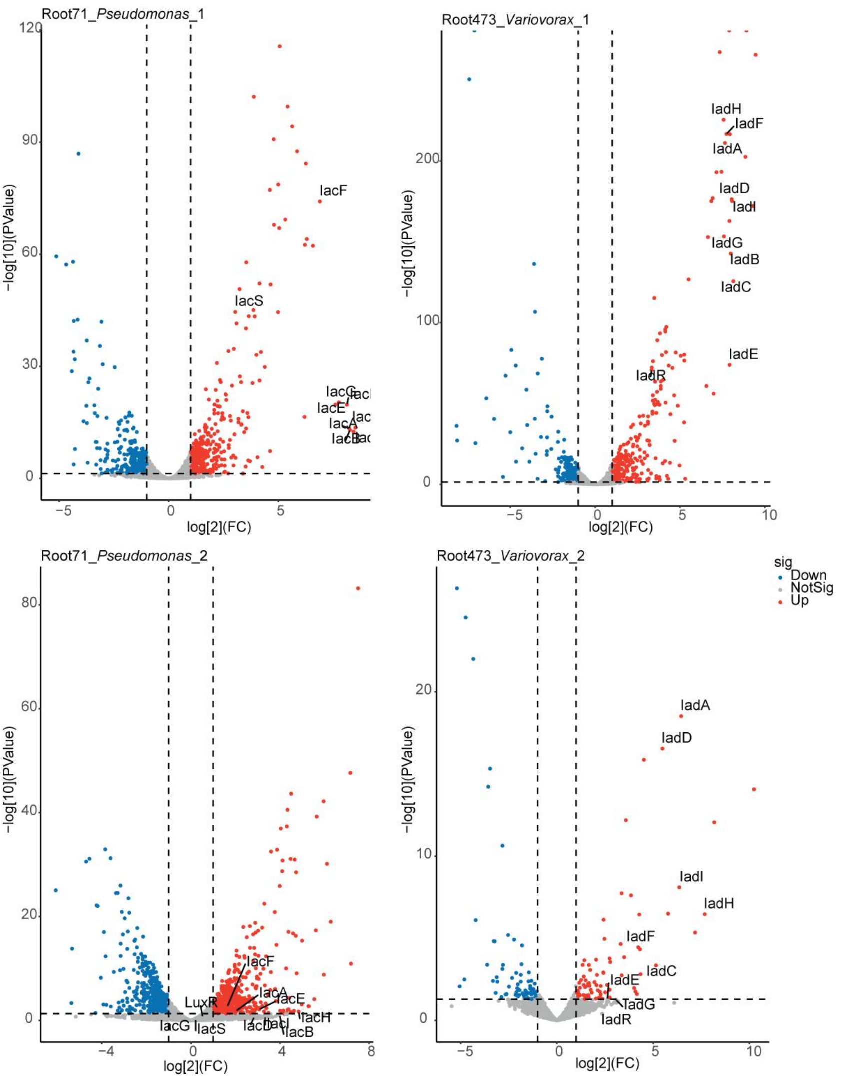

RNA-seq reveals that the transcription of the iac-like or iad-like gene cluster is induced by IAA. Root71_*Pseudomonas* containing iac-like operpon and Root473_*Variovorax* containing iad-like operon were selected to perform this experiment. Root71_*Pseudomonas*_1 and Root473_*Variovorax*_1 represent data from the first batch of RNA-seq experiment, where the bacteria were cultured in M9 medium supplemented with either IAA or glucose and ethanol. Root71_*Pseudomonas*_2 and Root473_*Variovorax*_2 are the data collected from the second batch of RNA-seq experiment, where the bacteria were cultured in M9 medium supplemented with either glucose and IAA or glucose and ethanol. The differential expression genes between IAA and Glucose treatments were analyzed. Significantly up-regulated or down-regulated genes were identified with $log_2^{FoldChange} \geq 1$ or ≤ -1 and adjusted *p*-value < 0.05 as cutoffs. Three individual colony of each strain were used to perform the transcriptome analyses.

Minor comments:

Point 2.5: l. 156 ff: The in vitro assay revealed 32 strains with considerable IAA degradation capability, but the genome screening resulted in 21 candidates. What does this mean with respect to presence or absence of the iac and iad gene clusters in those

other 11 strains?

Response: We further focused on the 11 strains which exhibited weak IAA degradation capabilities. None of these strains contain proteins predicted to be members of iac or iad-like operon (see **Supplementary Figure 3**). The degradation efficiency of these strains was tested, and compared to the beginning of the experiment, the IAA content in the medium was reduced by less than 30% (see **Figure1**). Thus we have revised this part as "**A total of 21 out of 183 strains displayed considerable IAA degradation efficiency, such that over 60% of the IAA content were consumed (Figure 1)** ."

Point 2.6: l. 202: Can the authors elaborate or speculate if the gene regulation via this two-component system would be any different from the previously identified regulator of the Pseudomonas genus?

Response: Thank you for your insightful question. The potential differences in gene regulation via the two-component system (TCS) identified in our study compared to the previously identified regulator of the *Pseudomonas* may be reflected in the following aspects.

The putative TCS consists of a sensor histidine kinase signal transducer-encoding gene (*iacS*) and a LuxR family protein receptor encoding gene (*LuxR*) ([4]). IacS senses IAA, autophosphorylates, and then phosphorylates LuxR, which positively interacts with the downstream gene promoter. According to a previous study, IacR acts as a repressor of *iacA* expression in *Pseudomonas putida* 1290 [5]. It may bind to the promoter region of *iacA*, and be released in the presence of IAA, thereby activating downstream gene expression [6].

Point 2.7: l. 264: "performed" instead of "preformed"

Response: Thank you for your comments, we have revised it.

Response to Reviewer #3:

Point 3.0: In this study, Wang et al. conducted a screening to identify bacteria capable of degrading the plant hormone auxin. The manuscript is clear and very easy to read. The methods employed are adequate and the conclusions are well supported by the results. The study addresses an important topic in microbiome research, namely the impact that plant-associated microbes have on plant hormonal metabolism. However, the findings are mainly confirmatory and do not significantly advance our comprehension of the biological role of auxin degradation by plant-associated bacteria. While the manuscript demonstrates that some bacteria can utilize indole-3-acetic acid (IAA) as the sole carbon source in liquid medium under controlled laboratory conditions, it falls short in explaining the significance of IAA degradation within the context of a plant-associated microbial community. Furthermore, the evaluation of the impact of auxin degradation on plants is limited to an assay that examines whether IAA-degrading bacteria (in monoassociation)

can suppress the effects of exogenous auxin on root growth in plants grown on agar plates. In summary, this is a well-executed study that confirms that some plant-associated bacteria can degrade auxin. Additional comments are provided below.

Response: We greatly appreciate the valuable feedback provided by the reviewer on our work. Below we provide a point-by-point response to the reviewer's comments.

Point 3.1: The authors propose that auxin degradation is a relevant trait in the plant microbiome. Some strains could suppress the RGI phenotype induced by exogenous auxin. In a natural scenario, what would the role of these strains be? What would be the source of "excess" auxin? I think that exploring these points would enrich the manuscript.

Response: Thank you for your comments. In nature, root tips and amount of plant associated microbes are natural source of auxin. Actually, we have tested the IAA biosynthesis ability of strains isolated from Arabidopsis root by adding trytophan into the medium and measuring IAA with Salkowski method (data not shown). Some of the tested strains have the ability to produce auxin. According to the result, we built a synthetic bacterial community by mixing 110 bacterial isolates. Inoculation with this synthetic community inhibit the primary root elongation in Arabidopsis, while RGI induced by this community inoculation was partial restored by adding IAA-degrading strains. We have add this part in result, and **Supplementary Figure 14** (<u>Please see the inserted figure in Point 2.1</u>). Please see line 311-338 in page 11. "**To further investigate the biological roles of IAA degraders in a natural context, RGI induced by exogenous IAA treatment was replaced with a synthetic bacterial community formed from 110 Arabidopsis root isolates (hereafter referred to as Syncom110). Fifteen strains of IAA-degrading bacteria isolated from Arabidopsis roots were grouped into different clusters to assess their ability to counteract RGI induced by Syncom110 (Supplementary Table 4). The primary root elongation was severely inhibited by inoculation with Symcom110. Interestingly, only clusters containing *Variovorax* strains were able to partially revert the RGI caused by Syncom110 (Supplementary Figure 14). These results are consistent with previous report** ".

Point 3.2: I am confused about the data displayed in the heatmap in Fig. 2b. Colors represent protein similarity (TM-score) in pairwise comparisons. Here, I would expect that the comparison "protein A vs protein B" would give the same score as "protein B vs protein A". However, this does not seem to be the case. For instance, "Root565 vs 7L19" has a score of ~0.5. On the other hand, "7L19 vs Root565" has a score of ~0.8. Why is this? Something seems off here. Please, clarify.

Response: TM-Score is the index of the structural similarity between two proteins. The formula to calculate the index as follow. $\text{TM-score}=\text{Max}\left[\frac{1}{L_N}\sum_{i=1}^{L_T}\frac{1}{1+\left(\frac{d_i}{d_0}\right)^2}\right]$ Where L_N is the length of the native structure, L_T is the length of the aligned residues to the template structure, d_t is the distance between the *i*th pair of aligned residues and d_0 is a scale to

normalize the match difference. ‘Max’ denotes the maximum value after optimal spatial superposition. The value of the TM-score always lies between (0,1], with better templates having higher TM-scores [7].

The sequence lengths of both proteins are considered during calculation, the score of “protein A vs protein B” is often not equal to “protein B vs protein A” due to the different sequence length of these proteins.

Point 3.3: Supplementary fig. 3c. Please, consider showing the LC-MS profile for all 21 strains, instead of only representative strains.

Response: Thank you for your comments, we have revised it. Please see the **Supplementary Figure 4**.

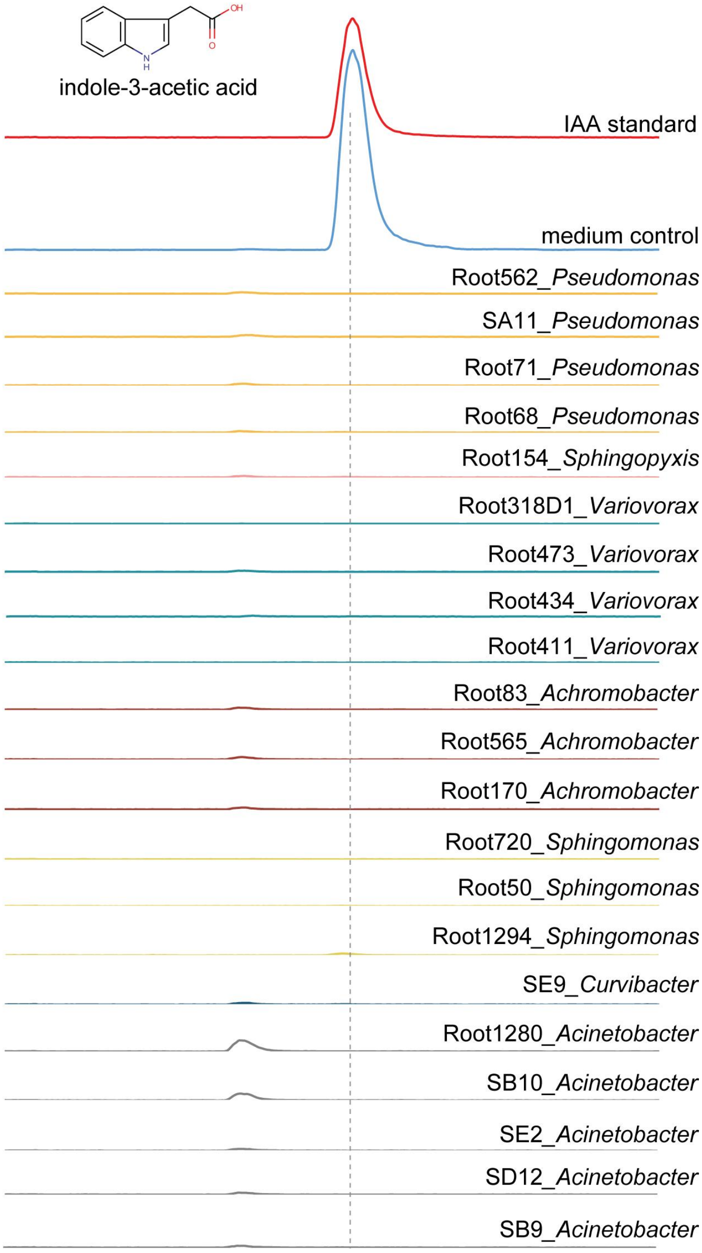

Supplementary Figure 4. LC-MS validation of IAA degradation in 21 strains.

Retention time of IAA in samples are consistent with commercial standard.

Point 3.4: Is the structural similarity sufficient to ascribe a role for the putative MaRs in auxin-binding/regulation? While I agree that, together with the genomic analysis, this seems strong evidence, the authors seem very certain, but there is no experimental evidence.

Response: Thank you for your insightful comment. We acknowledge that while structural similarity and genomic analysis provide strong indications, they do not constitute direct experimental evidence. Our intention was to highlight the circumstantial evidence supporting our hypothesis that the putative MarRs function in a similar way due to their similar protein structure. We have added a sentence at the end of this section to reflect a more cautious interpretation of the data. Please see line 235-237 in page 9. " **The conservation of protein structure and the IAA binding sites may elucidate why different types of MarRs exhibit similar mechanisms of action in regulating the expression of downstream gene clusters**. "

Point 3.5: Lines 273-275. Please, adjust the grammar in the following sentence: "…which generally degraded IAA completely within 60 hours, also showed extremely slow growth rate may suggest that cell proliferation of these strains require more carbon and energy source."

Response: Thank you for your comments, we have revised it.

Point 3.6: Line 298, replace "rest" by "remaining".

Response: Thank you for your comments, we have revised it.

Point 3.7: Please, consider incorporating the results of IAA degradation in Figure 1 (i.e., move it from the supplementary material).

Response: Thank you for your comments, we have revised it. Please see the new **Figure 1**.

Point 3.8: Line 122. "…the MarR family regulator was present in the operon of all genera with high similarity in their protein structures expect for Pseudomonas, which…". This sentence is confusing. The authors likely mean "except" instead of "expect".

Response: Thank you for your comment, we have revised it.

References

1. Kumar, S., Diksha, Sindhu, S.S., and Kumar, R. (2022). Biofertilizers: An ecofriendly technology for nutrient recycling and environmental sustainability. Current research in microbial sciences *3*, 100094.
2. Yan, Z., Chen, B., Yang, Y., Yi, X., Wei, M., Ecklu-Mensah, G., Buschmann, M.M., Liu, H., Gao, J., Liang, W., et al. (2022). Multi-omics analyses of airway host–microbe interactions

in chronic obstructive pulmonary disease identify potential therapeutic interventions. Nature microbiology *7*, 1361-1375.

3. Tintelnot, J., Xu, Y., Lesker, T.R., Schönlein, M., Konczalla, L., Giannou, A.D., Pelczar, P., Kylies, D., Puelles, V.G., Bielecka, A.A., et al. (2023). Microbiota-derived 3-IAA influences chemotherapy efficacy in pancreatic cancer. Nature *615*, 168-174.
4. Donoso, R., Leiva-Novoa, P., Zúñiga, A., Timmermann, T., Recabarren-Gajardo, G., and González, B. (2017). Biochemical and Genetic Bases of Indole-3-Acetic Acid (Auxin Phytohormone) Degradation by the Plant-Growth-Promoting Rhizobacterium Paraburkholderia phytofirmans PsJN. Applied and environmental microbiology *83*.
5. Scott, J.C., Greenhut, I.V., and Leveau, J.H. (2013). Functional characterization of the bacterial iac genes for degradation of the plant hormone indole-3-acetic acid. Journal of chemical ecology *39*, 942-951.
6. Conway, J.M., Walton, W.G., Salas-González, I., Law, T.F., Lindberg, C.A., Crook, L.E., Kosina, S.M., Fitzpatrick, C.R., Lietzan, A.D., Northen, T.R., et al. (2022). Diverse MarR bacterial regulators of auxin catabolism in the plant microbiome. Nature microbiology *7*, 1817-1833.
7. Zhang, Y., and Skolnick, J. (2004). Scoring function for automated assessment of protein structure template quality. Proteins *57*, 702-710.
