# Supplementary material for: Systematic characterization of plant-associated bacteria that can degrade indole-3-acetic acid
Source: PLoS Biol. 2024 Nov 26;22(11):e3002921. doi: 10.1371/journal.pbio.3002921 (PMC11630574; doi:10.1371/journal.pbio.3002921)
Supplement: Attachment — Submitted filename: Response to Reviewers20241003.docx [file pbio.3002921.s024.docx]

Response to Reviewers

Reviewer #2:

**Point 2.0**

With their new submission, Wang and colleagues have carefully addressed the reviewers' comments on the previously submitted manuscript and provided detailed responses to the questions. Additional in planta experiments and new computational analyses were performed that improve clarity and comprehensiveness of the study and relevance of the findings. While I appreciate a lot the effort, and the paper thoroughly dissects the potential of IAA degradation in root-associated bacteria, my major concern remains that there is still an overinterpretation of the results with respect to the biological role of bacterial IAA degraders in association with a plant host.

**Response:** We greatly appreciate the valuable feedback provided by the reviewer on our work. Below we provide a point-by-point response to the reviewer’s comments.

**Point 2.1**

In a new experiment, Arabidopsis plants were germinated and transferred to agar plates containing a diverse synthetic bacterial community of 110 isolates, plus either no other bacteria, or 15 IAA degraders, or sets of members of those 15. As a control, agar plates without the Syncom were used. There is root growth inhibition (RGI), i.e., decreased primary root length elongation, when the Syncom is present. The authors interpret this as a result of IAA produced by the Syncom, which is a very speculative claim. Nevertheless, the addition of some of the degraders causes suppression of RGI, which is quite interesting. This suppression by one or all of the 15 members of IAA degraders could be a consequence of several IAA-independent scenarios. Microbes antagonizing the ones that cause RGI, general root growth promotion by individual strains, etc. If the authors could show that indeed IAA concentration on the root/in the system in planta decreases, the evidence would be much stronger. Another option could be to generate a bacterial mutant that lacks the capability of IAA consumption or degradation and test if RGI suppression is abolished. These may be difficult experiments to establish. However, as long as direct evidence for the relevance of IAA degradation on the plant root is missing, it is impossible to conclude that RGI suppression is the biological role of IAA degraders. It is then crucial to be very careful in the phrasing and refrain from posing strong claims like "key role" and "reveal the biological role of IAA homeostasis".

**Response:** Thank you for your valuable comments. We acknowledge the claim regarding Syncom110-induced RGI being driven by IAA production is speculative. Additionally, drawing conclusions without substantial experimental evidence demonstrating that IAA-degrading bacteria indeed reduce IAA concentrations on the roots would be premature. Although additional experiments such as measuring *in planta* IAA concentrations or generating IAA degradation-deficient mutants would indeed strengthen our conclusions, as the editor has suggested, we believe that the current experimental design provides insights. Given the complexity and time required for the proposed additional experiments, we have opted not to pursue them at this stage. In response to this, we have revised our manuscript to more carefully frame our conclusions. Specifically, we now explicitly acknowledge the correlative nature of our findings and have removed statements that imply a definitive causal relationship between IAA degradation and RGI suppression. We have reworded phrases such as "key role" and "reveal the biological role of IAA homeostasis" to reflect the potential, but as yet unproven, involvement of IAA-degrading bacteria in the suppression of RGI. We also discuss alternative possibilities for RGI suppression, including direct interactions between strains and general growth promotion, as you suggested. We hope that these revisions provide a clearer, more balanced interpretation of our results while addressing your concerns. Here is the revised Discussion section, with emphasis on the highlighted parts. Please see line 498-534, page 16-17.

**“Plant growth requires multifaceted regulation, including but not limited to IAA production and degradation by plant, as well as auxin regulation by plant-associated microbiota [25]. It is estimated that 80% of rhizosphere commensal isolates possess the capability of producing IAA [15]. While, based on our results, only 11.48% of the isolates (21 out of 183 in this study) and 7.84% (131 out of 1465) of the plant-associated MAGs (S5 Table) exhibit potential IAA-degrading capability. The results of bacterial inoculation experiments suggest a potential role for IAA degraders in influencing IAA homeostasis in plants, potentially contributing to the maintenance of root growth. However, not all IAA-degrading strains have the ability to reverse the severe RGI induced by excess IAA or a synthetic bacterial community. Taking *Pseudomonas* as an example, the addition of IAA to the MS medium may have triggered the transcription of the *iac*-like operon, enabling IAA degradation and further partially alleviating RGI phenotype in Arabidopsis caused by exogenous IAA. However, in a more complex microbial community, introduced bacterial members may engage in direct antagonistic interactions with *Pseudomonas*, potentially preventing the transcriptional activation of *iac*-like operon due to a trade-off in the competitive dynamics. Additionally, *Pseudomonas* is also a notable producer of IAA. Studies have reported that IAA synthesis is a crucial mechanism enabling commensal bacteria to resist plant-generated reactive oxygen species (ROS) and establish successful colonization [14]. In the presence of competitors, *Pseudomonas* may activate its IAA synthesis pathway to enhance colonization on plant roots. These hypothesis based on the current research findings, though the precise molecular mechanisms remain to be further investigated.**

**Notably, inoculation with *Variovorax* not only restored root growth in Arabidopsis and rice induced by exogenous IAA, but also partially alleviated RGI caused by Synthetic bacterial community inoculation. This finding is consistent with recent study, and further validate the importance of *Variovorax* in plant root morphogenesis [24, 25]. *Variovorax* may assist plants in regulating IAA concentrations through its IAA degradation capability, thereby preventing RGI caused by excessive IAA in rhizosphere produced by other microbes. Additionally, compared to the axenic control, inoculation with *Variovorax* alone can promote primary root growth (S15 Fig). Co-inoculation with Syncom110 showed partial reversal of RGI, which may also be attributed to the general root growth-promoting effect of *Variovorax*. Moreover, members from this genus may be involved in the regulation of IAA signaling or interact with other plant hormones, such as cytokinins, which warrants further investigation.**

**Point 2.2**

Regarding the presentation of the data, I find the photos of the plants on agar plates a bit confusing when directly compared with the root length elongation data. The elongation is of course not visible in the plant photos. It may make sense to make this clearer either by saying explicitly in the figure caption that, although absolute root length of the plants may be similar between conditions, this is a consequence of differential root length elongation, or by indicating on the photos where the initial root tip was after transfer to the plates.

**Response:** Thank you for your insightful suggestion.

We have standardized the data presentation throughout the manuscript by calculating the fold change of the treatment (inoculation) relative to the control, followed by applying a log2 transformation to the resulting values. Please see Fig 5CD and S11A Fig and S15A Fig. We have marked the initial root tip positions with red arrows in the photos to clearly indicate where root elongation began. Please see Fig 5E and S11B Fig, S12 Fig and S15B Fig. We have explicitly mentioned in the figure captions as “Red arrows indicate the original positions of the root tips at the time of bacterial inoculation.”

Minor comments underlining the advice to thoroughly proof-read the manuscript with respect to grammar, phrasing, and accuracy:

**Point 2.3**

Salkowski method still appears in the text (e.g., line 153) and in the figures (e.g., Fig. 1).

**Response:** Thank you for your comments. We have replaced “Salkowski method” with “**colorimetric assay**”

**Point 2.4**

Revise grammar in Figure S11. Also, not clear if NB-IAA means with or without IAA.

**Response:** Thank you for your comments. We have revised the grammar in the figure legend and replaced “NB-IAA” with “**NB without IAA**”.

**Point 2.5**

Line 401-403: This sentence does not seem to be complete.

**Response:** Thank you very much for pointing the issue. We have added the verb “is” into the sentence. “**A complete iac-like operon is uniquely present in the genomes of strains from *Sphingomonas*.**”

**Point 2.6**

Line 422: suggesting instead of suggested

**Response:** Thank you for your comment, we have revised it.

**Point 2.7**

Line 521: What does half-strength TSB mean? How much g/L?

**Response:** Thank you for your question. “half-strength TSB” refers to Tryptic Soy Broth (TSB) prepared at half the standard concentration. The full-strength TSB is generally prepared at 30 g/L. Here we prepared the medium at 15 g/ L. We have clarified this in the manuscript as “**half-strength TSB (15 g/L)**” in line 624 page 20 to avoid any misunderstanding. We also clarified this upon the first mention of the term “TSB” on line 580, page 19, as “**^1^/_5_ tryptic soy broth (TSB, 6 g/L, Sigma-Aldrich, USA)** ”.

**Point 2.8**

Line 533: Please explain in a bit more detail what the Salkowski method is and what it visualizes.

**Response:** Thank you for your comment. The Salkowski method is a colorimetric assay used to detect and quantify the presence of IAA. In this method, IAA reacts with Salkowski reagent (a mixture of 0.5 M FeCl_3_ and 35% H_2_SO_4_), producing a color change that is typically pink due to IAA complex formation with and reduction of Fe^3+^. The color intensity at 530 nm is directly proportional to the concentration of IAA. This method enables both visual and quantitative assessment of IAA production (in medium supplemented with L-tryptophan) and IAA degradation (in medium supplemented with IAA) by microorganisms.

We have add more details in this section. “**Briefly, 120 μL of Salkowski reagent (a muxture of 0.5 M ferric chloride and 35% sulfuric acid) was mixed with 60 μL of culture supernatant. The residual IAA in the medium reacts with Fe^3+^ to form a pink compound, which exhibits an absorption peak at 530 nm. After 30 minutes of incubation in the dark, the absorbance was measured using a Synergy^TM^ H1 microplate reader.**”

**Point 2.9**

Line 575: Illumina

**Response:** Thank you for your comment. We have revised it.

----------------------------------------------------------------

Reviewer #3:

**Point 3.0**

This revised version of the manuscript successfully addresses most of the comments from my original review and is a clear improvement over the previous version. The addition of relevant new in planta experiments and results has strengthened the study, making it more comprehensive. I enjoyed reading the manuscript, and I believe the findings presented are valuable to the field.

The primary contribution of this study is its demonstration that bacteria capable of degrading auxin are relatively common within the plant microbiome. In this revision, the authors have responded to critiques by incorporating experiments that explore the in planta significance of auxin degradation by the root microbiome. Notably, they show that certain IAA-degrading strains can partially revert the root growth inhibition (RGI) phenotype induced by a synthetic community (Syncom110). Although the exact biological role of auxin degradation remains unclear, the study suggests that some of these bacteria may use indole-3-acetic acid (IAA) as a carbon source and could potentially influence root development. Thus, the study is primarily incremental and aligns well with and supports the conclusions published by Finkel et al. in 2020.

A few additional comments are provided below.

**Response:** We greatly appreciate the valuable feedback provided by the reviewer on our work. Below we provide a point-by-point response to the reviewer’s comments.

**Point 3.1**

I am still confused about the TM-score shown in Fig. 2B. If this is a measure of similarity between two protein structures, I would expect that 'A vs. B' would yield the same score as 'B vs. A'. However, this is not the case in this figure. As I am not an expert in protein structures, I cannot fully assess whether the analysis was done correctly. This lack of understanding raises a question: What is the actual result in this figure? What do we learn from it, given that the score varies depending on the direction of the comparison? The authors claim that IacR and IadR show high similarity to known MarR proteins, a conclusion I agree with based on Supplementary Figures 7 and 8. However, the presentation in Fig. 2B is confusing. What is the TM-score threshold for "high similarity"?

**Response:** We greatly appreciate the valuable feedback provided by the reviewer on our work. We also realize that the Fig 2B may lead some misunderstanding.

The structural similarity calculation using template modelling score (TM-score). We have supplemented the TM-score results in supplementary table 6. In this table, we displayed the TM-score1 (Left protein sequence as template ) and TM-score2 (upper protein sequence as template ). For example, as Root1294IacR2 and Root50IacR2share the same protein sequence length, resulting in identical TM-score for both TM-score1 and TM-score2 (0.90821). While, Root1294IacR2 has a different protein sequence length compared to Root154IacR2, leading to a difference between TM-score1 (with Root154IacR2 as the template) and TM-score2 (with Root1294IacR2 as the template).

A TM-score greater than 0.5 implies that two structures are likely to adopt the same fold and are evolutionary related . As show in the table, all pairwise TM-score is greater than 0.58, suggesting that these 27 MarR proteins share similar protein structures and are evolutionary related.

We recognize that the Fig 2B may cause confusion for readers. To address this, we have separated Fig 2B to create the new Fig 3. In Fig 3A, we present the lower TM-score in the heatmap. For example, the TM-scores between Root1294IacR2 and Root154IacR2 are 0.84168 and 0.8726, and we only display the lower value, 0.84168 in the heatmap. Additionally, we have selected representative structural models, including two template sequences (7KUA and 7KFO) and three MarRs from this study, and we displayed the TM-scores and RMSD values of these three proteins compared to the two templates in panel B. We hope this revised figure will provide clearer insights for readers.

**“To further elucidate the mechanism of functional conservation among MarRs, the predicted protein structures of 21 hypothetical MarRs were generated using AlphaFold2 [32] (Fig 3B, S8 Fig). The protein structures of six referential MarR proteins were retrieved from the PDB, and pairwise comparisons among the 27 MarRs were conducted by calculating their template modelling (TM) score [33, 34]. A TM-score greater than 0.5 implies that two structures are likely to adopt the same fold and are evolutionary related [35] As shown in the heatmap, all pairwise comparisons obtained a high TM-score (all pairwise TM-score > 0.58) (Fig 3, S6 Table), suggesting that all MarRs displayed high similarity in their protein structures.”** Please see Line 255-263 in page 9.

**Fig 3**. **Characterization of MarR protein structures.** (A), Protein structures of IacR or IadR (all belong to MarR family) are highly conserved among IAA-degrading strains. The heatmap displaying structural similarities among 27 MarR family proteins highlights the TM-score (the lower TM-score was displayed, all data was shown in Supplementary table 6) distribution. IacR and IadR were retrieved from 17 IAA-degrading strains in this study, along with 6 reference proteins whose protein structures and IAA binding sites have been identified. The phylogenetic tree was constructed using the IacR or IadR amino acid sequences. The IAA binding sites of MarR were conservatively distributed in IacR or IadR. Six MarR templates used in this analysis (labeled in red) are *Acinetobacter baumannii_*7L1I, *Pseudomonas putida_*7KUA, *Enterobacter soli* ATCC BAA-2102*_*7L19, *Bradyrhizobium japonicum_*7KYM, *Ruegeria pomeroyi* DSS-3_3CDH, *Variovorax paradoxus* CL14_7KFO. (B), Five selected representative structural models, including 7UKA, 7KFO, and three MarR proteins from distinct clusters, are displayed. The TM-score and RMSD (Root Mean Square Deviation) between the query and template are shown in the table on the right.

**Point 3.2**

Figure 4B shows the growth of the bacterium in M9 medium supplemented with IAA as the sole carbon source. The methods section indicates that M9 medium with ethanol (the IAA solvent) was used as a control. However, the result of the control condition is not clearly shown or used in the figure. Bacterial growth in medium with and without IAA should be presented side by side to better support the conclusion that some of these strains indeed use auxin as the carbon source.

**Response:** We appreciate the reviewer’s valuable comment. We have provided S10 Fig to include bacterial growth in both M9 medium supplemented with IAA and the control medium (M9 with 0.05% ethanol) side by side. This visual comparison better supports our conclusion that some strains utilize IAA as a carbon source. And we have also rewritten this section as follows. Please see Line 305-331 in page 11.

**“To evaluate the utilization of IAA by the IAA degraders, *in vitro* assays were performed among the screened 21 IAA degraders. The IAA degradation efficiency and bacterial growth of the strains were carried out in M9 minimal medium with exogenous IAA. In this study, 0.4 mM IAA with 0.05% ethanol for IAA solubilization was supplemented in the M9 minimal medium as sole carbon source. Our results showed that strains from *Acinetobacter* and *Pseudomonas* presented the maximum degradation and growth rates, consuming IAA completely within 12 or 24 hours (Fig 5A and B). This effect may be attributed to the ability of *Acinetobacter* and *Pseudomonas* strains to utilize ethanol to support initial population expansion, followed by rapid IAA degradation (S10 Fig). Interestingly, the growth of *Acinetobacter* strains were slightly inhibited in the presence of IAA, whereas IAA accelerated the growth of *Pseudomonas* (S10 Fig). On the other hand, other strains exhibited slower growth in M9 medium, which consequently impacted their IAA degradation rates. Notably, strains such as Root473_*Variovorax,* Root50_*Sphingomonas*, and Root83*_Achromobacter* exhibited accelerated growth after metabolizing IAA within 24 hours, indicating their ability to utilize IAA as a sole carbon source for growth. Finally, strains from *Variovorax*, *Achromobacter*, *Curvibacter,* *Sphingomonas* and *Sphingopyxis* exhibited either complete or partial IAA degradation within 72 hours, while also displaying extremely slow growth rate. This may suggest that the cell proliferation of these strains requires additional carbon and energy sources. The degradation of IAA observed in strains such as SE9_*Curvibacter* and strains from *Acinetobacter*, may serve alternative purposes, such as mitigating the toxicity of this compound, disrupting inter-microbial signaling, or facilitating their colonization on the root. Combined with the transcriptome analysis, our results here suggest that IAA triggers the expression of iac/iad-like operon and facilitates the biotransformation of this compound in the medium, even though IAA may not be suitable as sole carbon and energy source for cell growth. ”**

**S10 Fig. IAA-degrading strains can utilize IAA as carbon source.** Growth of individual IAA-degrading strains was measured at OD_600_ in M9 minimal medium supplemented with IAA (dissolved in ethanol) or M9 minimal medium supplemented with 0.05% ethanol as the sole carbon source (n=3). Significant differences compared between M9+IAA and M9+0.05% ethanol were determined using Student’s *t*-test: **P*<0.05, ***P*<0.01, ****P*<0.001.

**Point 3.3**

In Supplementary Figure 11, the first treatment is labeled "NB-IAA," which could be misinterpreted as "No bacteria and addition of IAA." The authors could prevent this misunderstanding by using a more explicit label, such as "NB without IAA."

**Response:** Thank you for your suggestion. We have revised the label in the S10 Fig. "NB without IAA."

**Point 3.4**

The discussion on the biological role of IAA degradation by plant-associated microbes is somewhat superficial. For instance, the authors mention that IAA-degrading bacteria have potential roles in regulating plant development. However, only one genus (Variovorax) could revert the root growth inhibition (RGI) phenotype induced by SynCom110 in a "natural" context. If only Variovorax can revert the RGI phenotype, what could be the role, if any, of the other IAA-degrading strains in plant development? More effectively highlighting and discussing the possibility that auxin degradation by the plant microbiome serves purposes yet to be discovered could set this paper apart from previous work on this topic.

**Response:** We appreciate your insightful comment. We have expanded the discussion to more thoroughly explore the biological role of IAA degradation by plant-associated microbes. Please see Line498-548 in page 16-18.

**“****Plant growth requires multifaceted regulation, including but not limited to IAA production and degradation by plant, as well as auxin regulation by plant-associated microbiota [25]. It is estimated that 80% of rhizosphere commensal isolates possess the capability of producing IAA [15]. While, based on our results, only 11.48% of the isolates (21 out of 183 in this study) and 7.84% (131 out of 1465) of the plant-associated MAGs (S5 Table) exhibit potential IAA-degrading capability. The results of bacterial inoculation experiments suggest a potential role for IAA degraders in influencing IAA homeostasis in plants, potentially contributing to the maintenance of root growth. However, not all IAA-degrading strains have the ability to reverse the severe RGI induced by excess IAA or a synthetic bacterial community. Taking *Pseudomonas* as an example, the addition of IAA to the MS medium may have triggered the transcription of the *iac*-like operon, enabling IAA degradation and further partially alleviating RGI phenotype in Arabidopsis caused by exogenous IAA. However, in a more complex microbial community, introduced bacterial members may engage in direct antagonistic interactions with *Pseudomonas*, potentially preventing the transcriptional activation of *iac*-like operon due to a trade-off in the competitive dynamics. Additionally, *Pseudomonas* is also a notable producer of IAA. Studies have reported that IAA synthesis is a crucial mechanism enabling commensal bacteria to resist plant-generated reactive oxygen species (ROS) and establish successful colonization [14]. In the presence of competitors, *Pseudomonas* may activate its IAA synthesis pathway to enhance colonization on plant roots. These hypothesis based on the current research findings, though the precise molecular mechanisms remain to be further investigated.**

**Notably, inoculation with *Variovorax* not only restored root growth in Arabidopsis and rice induced by exogenous IAA, but also partially alleviated RGI caused by Synthetic bacterial community inoculation. This finding is consistent with recent study, and further validate the importance of *Variovorax* in plant root morphogenesis [24, 25]. *Variovorax* may assist plants in regulating IAA concentrations through its IAA degradation capability, thereby preventing RGI caused by excessive IAA in rhizosphere produced by other microbes. Additionally, compared to the axenic control, inoculation with *Variovorax* alone can promote primary root growth (S15 Fig). Co-inoculation with Syncom110 showed partial reversal of RGI, which may also be attributed to the general root growth-promoting effect of *Variovorax*. Moreover, members from this genus may be involved in the regulation of IAA signaling or interact with other plant hormones, such as cytokinins, which warrants further investigation.**

**On the other hand, our mono-association results showed that *Acinetobacter* has negative effects on Arabidopsis growth (Fig 5, S12 Fig), which is inconsistent with recent reports that *Acinetobacter* can act as a plant growth-promoting rhizobacteria (PGPR) [46, 47]. In addition to being a strong IAA-degrading bacterium, *Acinetobacter* is also one of many that produce IAA and can thus sabotage plant physiology by adding to the endogenous IAA pool in plants [48]. This dual functionality may reflect the bacteria’ diversity or condition-dependent behavior in different habitat. Therefore, the biological role of *Acinetobacter* and its underlying mechanisms in plants require further investigation, particularly its specific position within the IAA regulatory network and its interactions with other plant hormones. Attention should be given to whether *Acinetobacter* exhibits different IAA metabolism strategies across various plant hosts or environmental conditions, in order to better understand its overall impact on plant physiology.”**

**Point 3.5**

Line 269: Replace "assay" with "assays."

**Response:** Thank you for your suggestion. We have revised the sentence by replacing “assay” with “assays”.

**Point 3.6**

Line 285: Replace "although" with "though."

**Response:** Thank you for your comment. We have revised sentence on by replacing “although” with “though” to clarify our conclusion.

.

**Point 3.7**

Line 294: Replace "additional" with the actual number of days in "After additional days of growth, primary root elongation was measured."

**Response:** Thank you for your comment. The sentence has been updated as “**After an extended growth period (7 days for Arabidopsis and 3 days for rice), primary root elongation was measured (see methods)**.” Please see line 360, in page 12.
